# Heat shock proteins and hormesis in the diagnosis and treatment of neurodegenerative diseases

**DOI:** 10.1186/s12979-015-0046-8

**Published:** 2015-11-04

**Authors:** Sandro Dattilo, Cesare Mancuso, Guido Koverech, Paola Di Mauro, Maria Laura Ontario, Cateno Concetto Petralia, Antonino Petralia, Luigi Maiolino, Agostino Serra, Edward J. Calabrese, Vittorio Calabrese

**Affiliations:** Department of Biomedical and Biotechnological Sciences, University of Catania, Via Andrea Doria, 95100 Catania, Italy; Department of Medical and Surgery Specialties, University of Catania, Catania, Italy; Department of Clinical and Experimental Medicine, School of Medicine, University of Catania, Catania, Italy; Institute of Pharmacology, Catholic University School of Medicine, Rome, Italy; Environmental Health Sciences Division, School of Public Health, University of Massachusetts, Amherst, MA USA; University College London Hospitals, NHS Foundation Trust, London, UK

**Keywords:** Alzheimer’s disease, Heat shock proteins, Heme oxygenase, Oxidative stress, Bilirubin, Neurodegenerative disorders, Vitagenes

## Abstract

Modulation of endogenous cellular defense mechanisms via the vitagene system represents an innovative approach to therapeutic intervention in diseases causing chronic tissue damage, such as in neurodegeneration. The possibility of high-throughoutput screening using proteomic techniques, particularly redox proteomics, provide more comprehensive overview of the interaction of proteins, as well as the interplay among processes involved in neuroprotection. Here by introducing the hormetic dose response concept, the mechanistic foundations and applications to the field of neuroprotection, we discuss the emerging role of heat shock protein as prominent member of vitagene network in neuroprotection and redox proteomics as a tool for investigating redox modulation of stress responsive vitagenes. Hormetic mechanisms are reviewed as possibility of targeted therapeutic manipulation in a cell-, tissue- and/or pathway-specific manner at appropriate points in the neurodegenerative disease process.

## Cellular stress response and the vitagene system

**Fig. 1:**
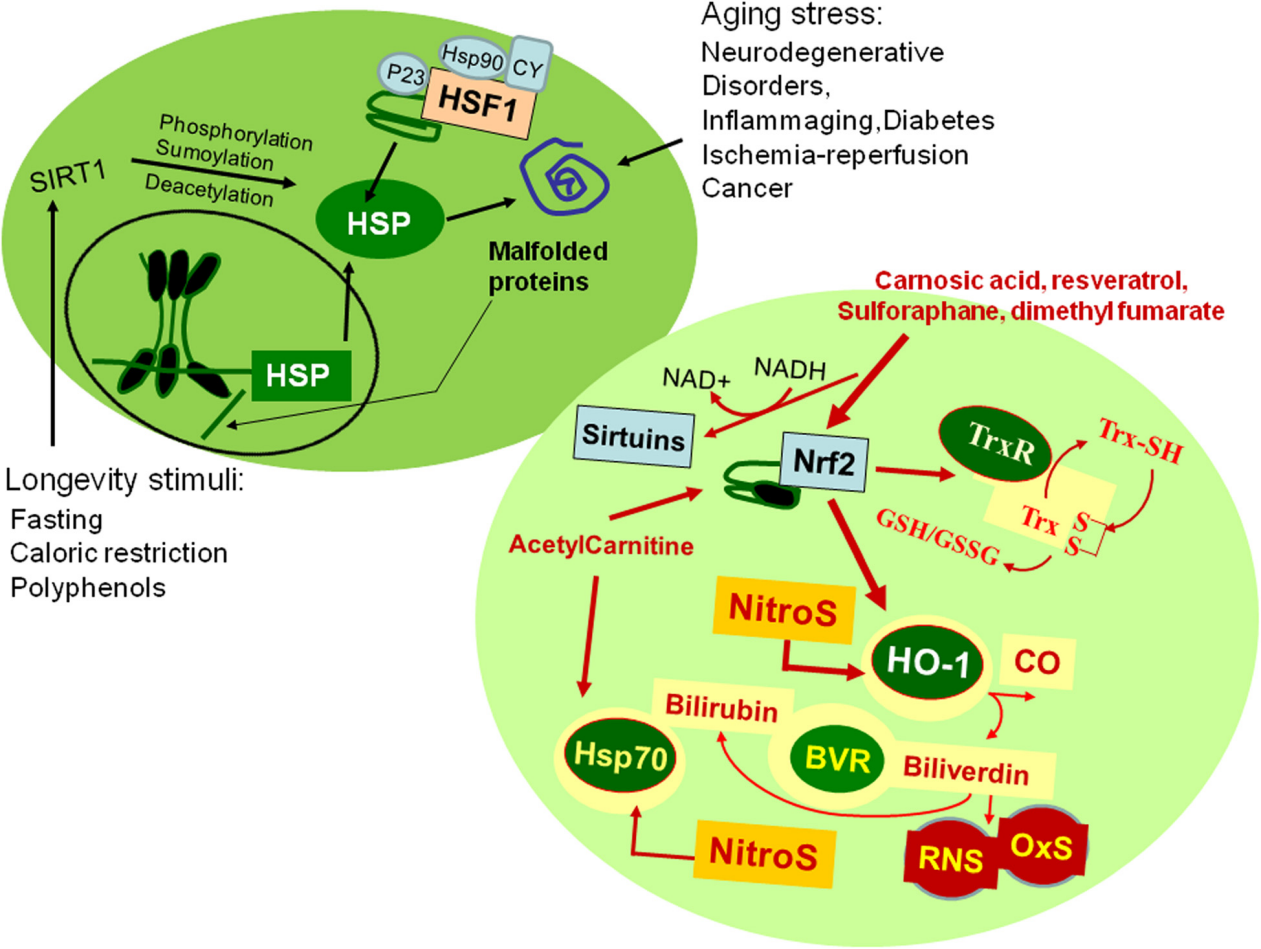
Vitagenes and the pathway of cellular stress response. Proteotoxic stresses causing accumulation of misfolded proteins trigger the cellular stress response. HSPs that are normally bound to HSF1 are titrate away by damaged proteins with resulting HSF-1 activation. Multi-step activation of HSF1 involves post-translational modifications, such as hyperphosphorylation, deacetylation or sumoylation, which allow HSF1 to trimerize, translocate into the nucleus, and bind to heat-shock elements (HSEs) in the promoter regions of its target *hsp* genes. Nutritional antioxidants, are able to activate vitagenes, such as heme oxygenase, Hsp70, thioredoxin reductase and sirtuins which represent an integrated system for cellular stress tolerance. Activation of Vitagene system, with up-regulation of HO-1, Thioredoxin, GSH and Sirtuin, results in reduction of pro-oxidant conditions. During inflammaging, including aged-associated pathologies a gradual decline in potency of the heat shock response occur and this may prevent repair of protein damage, leading to degeneration and cell death of critical parenchymal cells. Phytochemicals and acetylcarnitine act through the activation of transcription factor Nrf2, which after binding to the antioxidant responsive element up-regulates vitagenes

Protein thiols play a key role in redox sensing, and regulation of cellular redox state is a crucial mediator of multiple metabolic, signalling and transcriptional processes [1]. Under optimal conditions long-term health is maintained by protein homeostasis, a highly complex network of molecular interactions that balances protein biosynthesis, folding, translocation, assembly/disassembly, and clearance [2, 3]. Protein quality control is a critical feature of intracellular homeostasis [4]. When conformationally challenged aggregation-prone proteins are expressed, the resulting unfolded or misfolded proteins are rapidly degraded via the ubiquitin–proteasome pathway. The ability of a cell to counteract stressful conditions is also known as cellular stress response or heat shock response, which is an ancient and highly conserved cytoprotective mechanism [3, 5]. Production of heat shock proteins (HSP), including protein chaperones, is essential for the folding and repair of damaged proteins, serving thus to promote cell survival conditions that would otherwise result in apoptosis [6]. There is significant interest in the discovery and development of small molecules that modulate heat shock responses and parallel stress response pathways for therapeutic purposes [1, 7–9]. The cellular stress response is regulated at the transcriptional, translational and post-translational levels. The major regulator of the heat shock response genes is the heat shock transcription factor 1 (HSF1) which is kept in a latent state by an inhibitory complex of stress-proteins, and plays a key regulatory role in response to environmental stress, development, and many pathophysiological conditions, including cancer, ischemia-reperfusion injury, diabetes, and aging [10, 11] (Fig. 1).

Mammalian cells contain at least 3 HSF family members, HSF1, HSF2 and HSF4 [12, 13]. Neurons appear to be deficient in the heat shock response while retaining the ability to express such HSF proteins [14]. Furthermore, HSF1 fails to be activated in motor neurons even when these cells are microinjected with plasmids encoding an HSF1 expression vector, suggesting a block to the HSF1 signal transduction pathways [15]. HSF1 is repressed under non-stress conditions by a complex containing Hsp90 and other proteins. In this inactive state, HSF1 is a monomer that lacks the ability to bind cis -acting heat shock elements (HSE) in the promoters of HSP genes. Protein stress results in conversion of HSF1 from inactive monomer to DNA binding trimer and remodeling of the inhibitory molecular chaperone complex [16]. Activation of HSF1 by heat shock is a multi-step process, involving multiple inducible phosphorylation, dephosphorylation, acetylation and deacetylation steps, the sum of which results in the transcription of HSP genes. Extracellular signal input during heat shock involves tyrosine phosphorylation upstream of HSF1, involves the receptor tyrosine kinase HER2 and launches downstream signaling cascades through intracellular kinase Akt [17]. Akt regulates HSF1 at least in part by modulating its association with the phosphoserine binding scaffold protein [17].

The major activator of HSF1 is proteotoxic insults, like heat shock. Misfolded proteins displace HSF1 form the inhibitory chaperone complex, HSF1 trimerizes, becomes phosphorylated and is translocated to the nucleus where it is able to bind to the heat shock element of HSP genes [18, 19].

Cellular stress response requires the activation of pro-survival pathways as well as production of molecules endowed with anti-oxidant and anti-apoptotic activities, which is under control of protective genes called vitagenes [1, 4, 7]. Generally, molecular chaperones help hundreds of signaling molecules to keep their activation-competent state, and regulate various signaling processes ranging from signaling at the plasma membrane to transcription. In addition to these specific regulatory roles, recent studies have revealed that chaperones act as genetic buffers stabilizing the phenotypes of various cells and organisms [19]. This may be related to their low affinity for the proteins they interact with, which means that they represent weak links in protein networks [20]. Chaperones may uncouple protein, membrane, organelle and transcriptional networks during stress, which gives the cell additional protection. The same networks are preferentially remodeled in various diseases and aging, which may help us to design novel therapeutic and anti-aging strategies [21]. Among the cellular pathways involved in the so called “programmed cell life” and conferring protection against oxidative stress, a key role is played by the products of vitagenes [1, 4, 7, 22–25]. These include members of the HSP family, such as heme oxygenase-1 (HO-1), Hsp72, sirtuins and thioredoxin/thioredoxin reductase (Fig. 1) [7, 26, 27]. Heme oxygenase-1, also referred to as Hsp32, degrades heme, which is toxic if produced in excess, into free iron, carbon monoxide and biliverdin (BV) this latter being the precursor of bilirubin, a linear tetrapyrrole which has been shown to effectively counteract oxidative and nitrosative stress due to its ability to interact with NO and RNS [28, 29]. Sirtuins are a group of proteins linked to aging, metabolism and stress tolerance in several organisms [30, 31]. Mammalian sirtuins are histone deacetylases, requiring NAD^+^ as a cofactor to deacetylate substrates ranging from histones to transcriptional regulators. Through this activity, sirtuins are shown to regulate important biological processes, such as apoptosis, cell differentiation, energy transduction and glucose homeostasis [30]. Recent studies have shown that the heat shock response contributes to establishing a cytoprotective state in a wide variety of human diseases, including inflammation, cancer, aging and neurodegenerative disorders. Given the broad cytoprotective properties of the heat shock response there is now strong interest in discovering and developing pharmacological agents capable of inducing the heat shock response [1].

### Heat shock proteins and neuroprotection

In response to various forms of stress, cells activate a highly conserved heat shock response in which a set of HSP are induced, which play important roles in cellular repair and protective mechanisms [3, 32]. Evidence suggests that manipulation of the cellular stress response may offer strategies to protect brain cells from damage that is encountered following cerebral ischemia or during the progression of neurodegenerative diseases [1, 27, 33, 34]. Heat shock proteins are evolutionarily conserved and present in all cellular compartments. Some of the major chaperones (Hsp70, Hsp90, small Hsps) are present at high concentrations in non-stressed cells reaching 1–5 % of total cellular protein, consistent with an important role for chaperones in cellular homeostasis. Indeed, chaperones display various activities in the cell, such as (i) proper folding of nascent polypeptide chains, (ii) facilitating protein translocation across various cellular compartments, (iii) modulating protein activity through stabilization and/or maturation to functionally-competent conformation, masking mild mutation at the conformational level (iv) promoting multiprotein complex assembly/disassembly, (v) refolding of misfolded proteins, (vi) protecting against protein aggregation, (vii) targeting irreversibly damaged proteins to degradation, (viii) sequestering damaged proteins [35, 36].

Heat shock proteins are classified according to their molecular weight [37]. The 70 kDa family of stress proteins is one of the most extensively studied. Included in this family are HSC70 (heat shock cognate, the constitutive form), HSP70 (the inducible form, also referred to as HSP72) and GRP-75 (a constitutively expressed glucose-regulated protein found in the endoplasmic reticulum) [5]. Heat shock proteins 70 function in co- and post-translational folding and the quality control of misfolded proteins [38]. More specifically, HSP70 participate in folding and assembly of newly synthesized proteins into macromolecular complexes; aggregation prevention; dissolution and refolding of aggregated proteins; as well as protein degradation [20]. Heat shock proteins 70 have an N-terminal ATP-binding domain (NBD) and a C-terminal substrate-binding domain (SBD) which are both critical for chaperone function. Non-native substrates with exposed hydrophobic stretches within an accessible polypeptide backbone associate transiently with HSP70 via its SBD. ATP binding to the NBD triggers opening of the SBD binding pocket, decreasing affinity for polypeptide substrates, thereby accelerating both on and off rates. Reciprocally, substrate binding induces ATP hydrolysis, ‘closing’ the SBD and thus stabilizing the substrate-HSP70 complex [20]. It is this cycle of rapid but controlled binding and release of the substrate that fosters folding and assembly with partner proteins while preventing aggregation of substrates; however, detailed mechanistic understanding of how HSP70 accomplishes these feats is not yet available [20]. Numerous hypotheses have been put forth to explain the molecular mechanism of HSP70-induced structural conversion of substrate proteins. For example, an ‘entropic pulling’ mechanism has been proposed, whereby HSP70 binding stabilizes peptide segments in an unfolded state, causing local unfolding, thereby facilitating disaggregation and allowing refolding upon HSP70 release [39]. Co-factors, such as the nucleotide exchange factors (NEFs) and co-chaperones, are crucial regulatory components of the HSP70 cycle that confer versatility and specificity to the HSP70 chaperone machine [20]. The HSP40 co-chaperone targets substrates to HSP70 while stimulating ATP hydrolysis; NEFs like Bag-1 (BCL2- associated athanogene 1) and HSP110 reinitiate the HSP70 cycle by facilitating ADP release and rebinding of ATP [40]. Moreover, Bag-1 has the additional ability to bind to the 26S proteasome and another BAG isoform, the Bag-3 co-chaperone, links HSP70 to the macroautophagic degradation pathway during aging [17]. CHIP (carboxy terminus of HSC70-interacting protein), a co-chaperone of HSP70 that also has E3 ubiquitin ligase activity, cooperates with Bag-1, and possibly Bag-3, in order to facilitate degradation of terminally misfolded substrate proteins. Notably, mutations in HSP70 co-factors are lethal [41] or may be associated with neurodegenerative disease [41, 42].

Recent studies indicate that the heat shock response declines in aging cells and becomes weaker as organisms live beyond the mature adult stage [17]. Cells lose the capacity to activate the transcriptional pathways leading to HSP synthesis (Fig. 1). In neuronal tissues, decline in protein quality control has been widely predicted, as the etiology of a number of diseases involve aggregation-prone proteins that form inclusion bodies whose occurrence is linked to pathology. Heat shock protein 70 has been extensively implicated in the pathogenesis of misfolding disease [17]. Numerous studies indicate that HSP70 and components of the ubiquitin–proteasome system associate with inclusion bodies/plaques characteristic of misfolding diseases, indicating a general activation of the cellular quality control machinery in an attempt to circumvent the accumulation of misfolded species [43]. In these conditions the HSP70 system is unable to refold disease-related proteins, causing perturbation of protein homeostasis associated with disease onset. Several hypotheses account for this apparent disruption of the balance between the production of misfolded proteins and HSP70 activity. As misfolded disease proteins accumulate, these can overwhelm the capacity of the HSP70 system to control the cellular folding milieu [32]. Progressive reduction in protein levels and/or activity of HSP70 and other components of the quality control network may exacerbate this imbalance, permitting further accumulation of toxic misfolded proteins. Such reduction could be due to the ageing process, as transcription of HSP70 decreases during ageing of the human brain. Alternatively, disease processes themselves might cause, or worsen, chaperone deficiency. Inclusions have been proposed to sequester HSP70 and other proteins in a non-functional state, inhibiting their essential function in cellular processes [20].

Another studies about diabetes DPN is unrelated to one specific misfolded protein aggregate, In this case hyperglycemia can promote the oxidative modification of amino acids [44] that may impair protein folding [45], decrease mithochondrial protein import [46], and promote mitochondrial dysfunction [47].

The availability of transgenic animals and gene transfer allowing over-expression of the gene encoding for HSP70, has revealed that overproduction of this protein leads to protection in several different models of nervous system pathology [4]. Overexpression of HSP70 and/or its co-chaperones suppresses aggregation and toxicity in models of misfolding disease [48]. Increased HSP70 levels caused reduced aggregation and toxicity of tau and Aβ, respectively, two components associated with Alzheimer’s disease [5]. Similarly, overexpression of HSP70 reduces toxicity and accumulation of α-synuclein in high molecular weight and detergent-insoluble deposits [5]. Increased expression of HSP70 has been reported to be associated with a decrease in apoptotic cell death, an increase in the expression of the antiapoptotic protein Bcl-2, a suppression of microglial/monocyte activation, and a reduction in matrix metalloproteinases. Up-regulation of HSP70 likewise reduced apoptosis and the formation of co-aggregates of the prion disease protein, PrP [4]. Numerous studies have also shown that HSP70 overexpression reduces polyQ toxicity. Results obtained in vitro have elucidated the mechanism of action of HSP70 against misfolding and thus toxicity of disease proteins. Purified HSP70 acts preferentially on monomers or oligomers, rather than fibrillar aggregates of Aβ, huntingtin, and α-synuclein species modulating the aggregation process [4]. Heat shock proteins 70 inhibits the aggregation of Aβ and α-synuclein species even at substoichiometric levels, suggesting that HSP70 can recognize multimeric protein assemblies [20]. As mentioned before, the effect of HSP70 on aggregation, which requires its ATPase activity, is enhanced by the cochaperone HSP40. Thus, HSP70 together with HSP40 stabilizes huntingtin in a monomeric conformation and prevents accumulation of spherical oligomers which are the toxic species for fibril formation [49]. As a result, mutant huntingtin is deviated from the potentially toxic, fibrillar aggregation pathway and instead accumulates in amorphous aggregates, or other benign conformers. Sequestered in these conformers, mutant huntingtin may no longer participate in heterotypic interactions known to inactivate essential cellular machinery, such as polyQ-containing transcription factors [49, 50].

Following focal cerebral ischemia, HSP70 mRNA is synthesized in most ischemic cells except in areas of very low blood flow, due to scarce ATP levels. Heat shock proteins 70 are produced mainly in endothelial cells, in the core of infarcts in the cells that are most resistant to ischemia, in glial cells at the edges of infarcts and in neurons outside the areas of infarction [51]. It has been suggested that this neuronal expression of HSP70 outside an infarct can be used to define the ischemic penumbras, which means the zone of protein denaturation in the ischemic areas, consistently in in vivo transgenic mice overexpressing HSP70, compared to wild-type mice in a middle cerebral artery occlusion model of permanent cerebral ischaemia, it has been demonstrated that overexpression of HSP70 reduces the overall lesion size and also limits the tissue damage within the lesion [51]. Heat shock protein 70 overexpression in post-mortem cortical tissue of AD patients and an increase in HSP70 mRNA were found in cerebellum hippocampus and cortex of AD patients during the agonal phase of the disease [4, 37]. Consistently, the use of agents that limit microglial activation and inflammation in AD has recently emerged as an attractive therapeutic strategy for this disease. For instance, the vasoactive intestinal peptide (VIP), has been shown to prevent Abeta-induced neurodegeneration, through inhibition of major pathways involved in the production of inflammatory mediators, such as the p38 MAPK, p42/p44 MAPK, and NFkB cascades, in activated microglial cells [52]. In keeping with this, HSP70 induces IL-6 and TNFα in microglial cells in a mechanism which has been demonstrated to be NFkB and p-38 MAPK-dependent [53] and this leads to an increased phagocytosis and clearance of Aβ.

A large body of evidence now suggests a correlation between mechanisms of nitrosative stress and HSP induction. We have demonstrated in vitro and in vivo that cytokine-induced nitrosative stress is associated with an increased brain synthesis of HSP70 stress proteins. The molecular mechanisms regulating the NO-induced activation of heat-shock signal seems to involve cellular oxidant/antioxidant balance, mainly represented by the glutathione status and the antioxidant enzymes [1, 54, 55].

#### Neuroprotective effects of extracellular heat shock proteins

Heat shock proteins are transferred between cell types in the nervous system. Thus, stress tolerance in neurons is not solely dependent on their own HSP, but can be supplemented by additional HSP transferred from adjacent glial cells. Therefore, supplying exogenous HSP at neural injury sites could be an effective strategy to maintain neuronal viability. This idea has been tested in a number of model systems. Injection of HSC/HSP70 into the vitreous chamber of the eye protected retinal photoreceptors from photodamage. Application of exogenous HSC/HSP70 to the cut end of the sciatic nerve reduced cell death in sensory and motor neurons. Extracellular HSP70 protected spinal cord motor neurons deprived of trophic support *in vitro* or undergoing cell death *in vivo.* Thus, exogenous application of HSP has potential as a therapeutic strategy for acute injury in the nervous system. Heat shock proteins are released into the blood stream after stressful stimuli and this may represent an important feature of the stress response. Exercise stress has been reported to induce the release of HSP70 from the human brain into the blood stream *in vivo.* The biological significance of this neural release is yet to be determined [56, 57]. In addition, stress proteins, such as HSP90 are necessary for the maturation of several transcription factors, including the nuclear hormone receptors and the hypoxia-inducible factor-1 [58]. Remarkably, there is increasing interest in the interaction between Hsp90 and p53 [59], the latter being a transcription factor regulating apoptosis, cell cycle arrest, senescence, DNA repair and genetic stability, and is activated primarily during cellular responses to DNA damage [60]. Consistently, a large number of proteins need the help of molecular chaperones to maintain their activation-competent conformation. ‘Conventional’ inhibitors interact with their target, directly inhibiting its function. However, chaperone-based inhibitors do not interact with the effector proteins, but inhibit the ability of the associated chaperone(s) to maintain their activation-competent conformation. As a result, the client proteins became degraded by the proteasome [38]. Due to its biological relevance in the folding, maturation and stabilization of pro-tumorigenic client proteins, HSP90 is emerging as therapeutic target in cancer treatment, representing a viable drug target for the design of chemotherapies [38].

### The heme oxygenase family

The heme oxygenase (HO) isoforms have been recognized as dynamic sensors of cellular oxidative stress and modulators of redox homeostasis throughout the phylogenetic spectrum. Two main isoforms of heme oxygenase were identified so far and named HO-1 and HO-2 [61, 62]. Heme oxygenase-1 is the inducible isoform and it is overexpressed under conditions of oxidative/nitrosative stress whereas heme oxygenase-2 is the constitutive enzyme and it plays a main role in the physiologic turnover of cellular heme [61, 62]. Interestingly, although the constitutive nature, HO-2 (no HO-1!!!!) is also up-regulated as an effect of some drugs, such as glucocorticoids, opiates and nitric oxide [61–63]. Though this different behavior in terms of regulation, both HO-1 and HO-2 catalyze the same reaction, namely the oxidation of the alpha-meso-carbon bridge of the heme moieties of hemoproteins thus generating equimolar amounts of ferrous iron, carbon monoxide (CO) and biliverdin (BV) [62, 64].

**Fig. 2:**
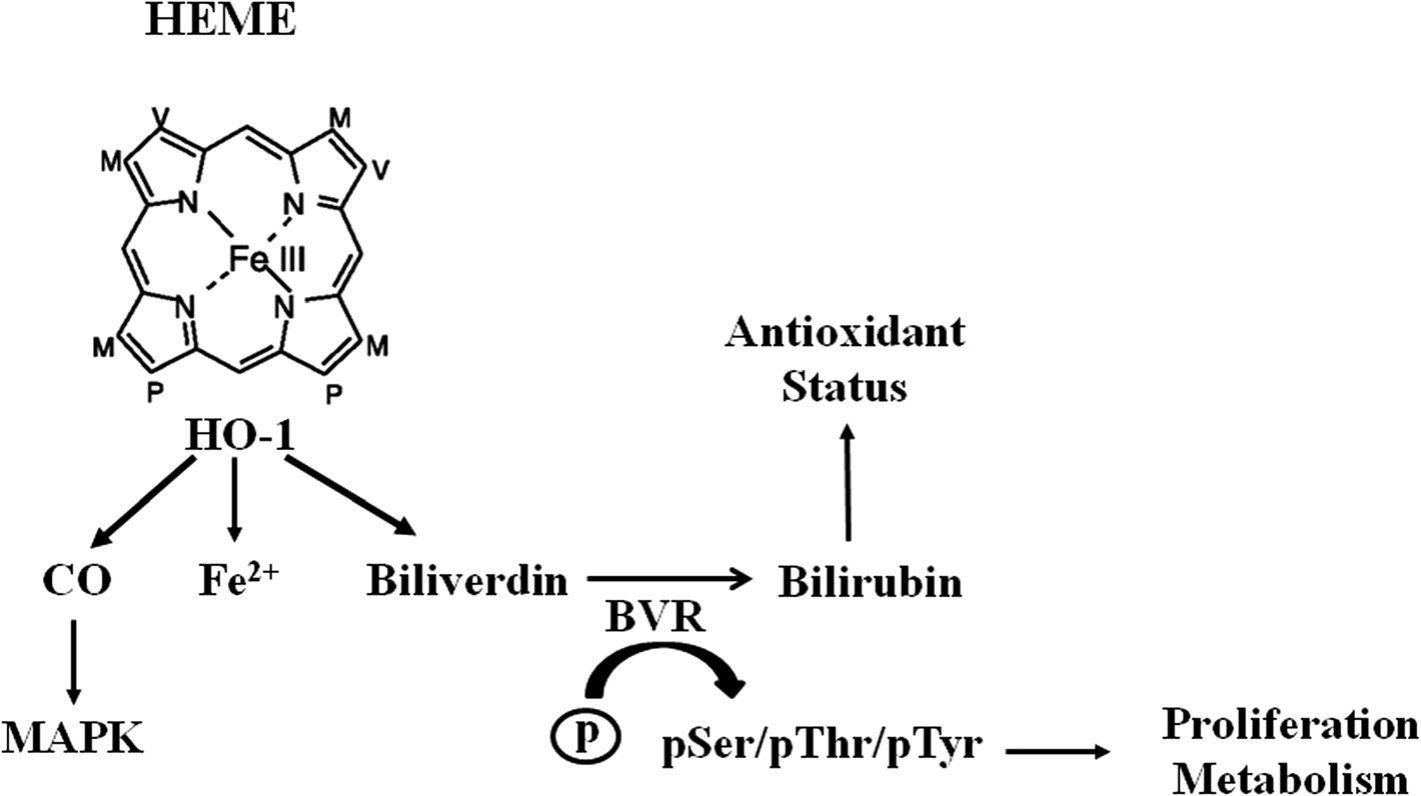
Heme metabolism and HO-1 enzyme activity. HO-1 catalyze the rate-limiting step in heme metabolism. Heme is cleaved by HO-1 to yield equimolar quantities of iron, CO, and biliverdin. The regulatory actions of CO can be, at least in part mediated, by the activation of MAPK pathway. Biliverdin is converted to bilirubin by biliverdin reductase, this latter being also endowed with Ser/Thr/Tyr kinase activity through which regulates cell growth and metabolism. At low physiological concentrations, bilirubin behaves as a powerful antioxidant

Among these by-products of HO activity (Fig. 2), CO is mainly involved in the regulation of important functions such as neurotransmission, regulation of neuropeptide release and modulation of the local/systemic immune-inflammatory response [65–68]. As far as BV concerns, it is a “virtual” by-product in mammal cells, because it is rapidly reduced to bilirubin (BR) by the cytosolic biliverdin reductase (BVR) [62]. Worth of noting is the fact that HO and BVR have to be considered as a single “fighter” against free radicals because of they reach the maximum of the antioxidant power when they act in concert. Over the last years, the role of BVR in the adaptive stress response was under-estimated because this enzyme was only considered necessary for the production of BR, which is responsible for free radical scavenging (Fig. 2). Several lines of evidence underlined the role of the HO/BVR system in the central nervous system and in neurodegenerative diseases. Previous preclinical studies put forth the idea of the neuroprotective role of the HO/BVR system because of its ability to (i) restore redox imbalance [2, 69], (ii) interact with neurotrophins [70], (iii) nitric oxide [71] and (iv) trap both reactive oxygen and nitrogen species [72–75]. However, recent results obtained in post-mortem brain tissues of subjects with Alzheimer’s disease (AD) demonstrated a down-regulation of both HO-1 and BVR in cognitive the hippocampus, a brain area involved in cognitive function. The down-regulation of these enzyme was secondary to phosphorylative, oxidative and/or nitrosative post-translational modification on the protein structures [75, 76]. These post-translational modifications are responsible for a reduced production of bilirubin and impaired interaction of BVR with members of the MAPK family [75]. A first corollary of these results is that the HO-1/BVR axis is not able to fully protect hippocampus in AD individuals. A possible approach to overcome this limitation in the neuroprotective activity is to prevent the oxidative and nitrosative modifications occurring on both HO-1 and BVR thus restoring their antioxidant potential. Very recently, atorvastatin, a well known hypolipidemic drug was shown to reduce oxidative/nitrosative stress biomarkers in the parietal cortex of aged canine, which is currently considered as the best predictive preclinical model to study dementia of Alzheimer type [77]. In particular, atorvastatin (80 mg/day for 14.5 months) was able to prevent oxidative/nitrosative modification on both HO-1 and BVR in the parietal cortex of aged canine [78, 79]. Importantly, atorvastatin increased BR production in the parietal cortex and improved cognitive skills in aged beagle dogs [79]. Taken together, these results demonstrate that the HO-1/BVR axis can be modulated by atorvastatin and open new avenues in terms of activation of the HO-1/BVR system and neuroprotection [80, 81].

### Hormesis

**Fig. 3:**
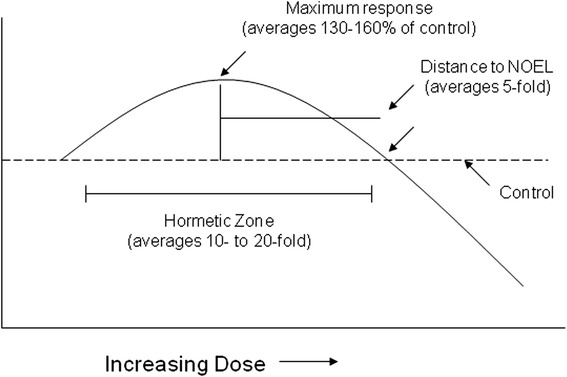
Dose–response curve depicting the quantitative features of hormesis

Hormesis is the most powerful endogenous protective mechanism against life threatening ischemic and oxidative insults to multiple organ systems [82]. It is a dose response phenomenon characterized by a low dose stimulation and a high dose inhibition (Fig. 3), that may be graphically represented by either an inverted U-shaped dose response or by a J- or U-shaped dose response. The term hormesis was first presented in the published literature in 1943 by Southam and Ehrlich who reported that low doses of extracts from the Red Cider tree enhanced the proliferation of fungi with the overall shape of the dose response being biphasic. However, credit for experimentally demonstrating the occurrence of hormesis goes to Hugo Schulz [83] who reported biphasic dose responses in yeast following exposure to a large number of toxic agents. The work of Schulz inspired a large number of investigators in diverse fields to assess whether such low dose effects may be a general feature of biological systems. In fact, similar types of dose response observations were subsequently reported by numerous researchers assessing chemicals [84] and [85–93] with investigators adopting different names such as the Arndt-Schulz Law, Huppe’s Rule, and other terms to describe these similar dose response phenomena.

Hormesis concept had a difficult time being incorporated into routine safety assessment and pharmacological investigations, principally because it (i) required more rigorous evaluation in the low dose zone, (ii) failure of investigators to understand its clinical significance (iii) failure to appreciate the quantitative features of the hormetic dose response (iv) failure to understand the limitations of its implications for commercial applications in agricultural as well as medicine, (v) because of the predominant interest in responses at relatively high doses during most of the 20^th^ century as well as (vi) the continuing, yet inappropriate, tendency to associate the concept of hormesis with the medical practice of homeopathy [94–97]. However, from the late 1970’s [98] there has been a growing interest in hormetic-like biphasic dose responses across the broad spectrum of biomedical sciences. This resurgence of interest resulted from a variety of factors, including the capacity to measure progressively lower doses of drugs and chemicals and the adoption of cell culture methods which has permitted more efficient testing. Re-examine the validity of linear at low dose modelling of cancer risks due to their enormous cost implications for regulations [99, 100] as well the astute observations of independent investigators and their capacity to generalize their findings across biological systems [98, 101].

What has emerged from these research initiatives from highly diverse biomedical areas is the recognition that hormetic dose responses were common and highly generalizable, being independent of biological model, endpoints measured and chemical class and/or physical agent studied [84, 85, 92, 102, 103]. This was an unexpected finding as hormetic responses were often considered by many in the so-called mainstream branches of toxicology and pharmacology to be paradoxical, not commonly expected and being of questionable reliability with a lack of capacity for replication. The casual dismissal of the hormesis concept during the mid decades of the last century is reflected in the general absence of the hormesis concept from the leading toxicological and biomedical textbooks. This situation has radically changed such that hormesis is now incorporated into all leading textbooks of toxicology [104] encyclopedias [105–107] and other leading monographs. In fact, while the terms hormetic and hormesis were cited only about 160 times during the entire decade of the 1980’s within the Web of Science database, in 2014 alone these terms were cited over 6,000 times.

Of further significance were observations that these broad ranging dose response relationships also shared the same general quantitative features. More specifically, the low dose stimulation which becomes manifested immediately below the pharmacological and toxicological thresholds is modest in magnitude being at most only about 30–60 % greater than the control group response. The width of the hormetic stimulation is usually about 10–20 fold starting immediately from the zero equivalent dose (i.e., estimated threshold) (Fig. 3). The hormetic dose response may result from either a direct stimulation or via an overcompensation stimulatory response following disruption in homeostasis [107, 108]. Regardless of the mode of action by which the stimulation occurs the quantitative features of hormetic dose responses are similar. These observations are based on copious data derived from the published literature ranging from plants to humans [109, 110], involving numerous receptor systems [111, 112]. These findings have led to nearly 60 biomedical scientists recommending that biological stress responses, including those of pre- and post-conditioning, be integrated within an hormetic context, along with the adoption of a terminology that would be based within an interdisciplinary framework [113].

The hormetic dose response confers a new set of interpretations for the dose response. At high doses within a toxicological setting, the typical endpoints measured indicate cellular damage. However, as the dose decreases below the threshold the low dose stimulation more likely represents a manifestation of an adaptive response that conforms to a measure of biological performance as may be seen in the cases of modest increases in cognition, growth, longevity, bone density and other biomedical endpoints of interest. The consistency of the vast array of hormetic findings suggests strongly that this dose response may be a manifestation of the plasticity of biological systems. Numerous papers have explored how hormesis may affect aging and numerous diseases associated with age including cardiovascular disease and a range of neurodegenerative conditions and their underlying mechanisms [114–118]. How the hormesis concept may prolong life and reduce the occurrence of chronic disease involves the optimized challenging of cells and whole organisms by any of a wide range of stressors including pharmacological, physical, dietary, exercise, and ischemic. Using a wide variety of in vitro and in vivo models, anti-aging and neuroprotective effects have been reported using hormetic experimental protocols [119–122]. Essentially all biological models respond to imposed stress with the same quantitative features of the dose response is a central finding within the biological sciences that has not been previously recognized. These findings suggest that the hormetic dose response would have been broadly selected for and highly conserved. This adaptive response not only enhances survival by conferring resistance to environmental stress but it represents a way to regulate the allocation of biological resources in a manner that ensures cellular and organismal stability.

These quantitative features of the hormetic dose response have important medical implications. Most significantly, the hormetic dose response imposes constraints upon the magnitude of a drug to induce a desired effect. For example, if a drug increased cognitive performance in an elderly patient by approximately 25–30 %, the hormetic model suggests that this level of performance could not be further increased using a new drug combination. This concept has been supported in a variety of studies on hormesis and drug interaction. Flood [123, 124] has demonstrated that the hormetic response for memory was bounded by the 30–60 % increase even when several drugs were used in combination which were designed to maximize memory outcome. This response magnitude constraint has been reported for immune stimulation, bacterial growth, increases in hair growth, plant growth, decrease in anxiety, decreases in tumor incidence and numerous other endpoints [125].

This limitation in the magnitude of the stimulatory response is a critical implication of the hormesis dose response concept. It is an observation which is based on extensive findings and it is a controlling feature which defines what pharmaceutical companies can expect to achieve with drugs that are designed to enhance performance. However, the limitation in the magnitude of response is also potentially important with respect to the capacity to detect a desirable response. This may not be a particularly important issue when using highly inbred animal models or cell cultures where experimental conditions can be highly controlled. However, attempting to measure a low dose hormetic stimulation within the context of a clinical trial can be problematic. Given the likelihood of considerable human variation in response to a drug, it is possible that the test population may have their responsiveness distributed over a range of responses that includes toxicity, optimal response and a group in which the dose is ineffective. The data from all subjects in such studies would normally be averaged together leading to a marked dilution of an overall positive treatment effect in the optimal response zone subgroup. This suggests a possible reason why drugs that were very successfully tested in preclinical studies with highly inbred strains of animals could and often have failed during the clinical trial. Of particular note is that investigators may have to modify doses based upon the sensitivity or susceptibility of the subjects. Calabrese and Baldwin [126] have shown that the hormetic dose response is often expressed in the broad range of subjects independent of their susceptibility. As expected, those individuals that are very resistant to the drug or chemical treatment would have their hormetic response shifted to the right on the dose response graph whereas those individuals with greater than normal susceptibility would have their hormetic response shifted to the left. The hormetic dose response therefore imposes considerable challenges to the biomedical community that is interested in the development of drugs that are concerned with improvements in human performance.

The hormetic dose response can also have undesirable effects. This may be most readily seen in the case of drugs that are designed to suppress growth or kill cells or organisms at higher doses. For example, there is now substantial evidence that low doses of many antitumor drugs can stimulate the proliferation of such cells at lower concentrations [127]. This also been shown to be the case with antibiotics, including penicillin [128] and streptomycin [128–130]. This phenomenon has also been reported with selected cardiac glycosides that have effects on non-target tissues such as the prostate where it is able to enhance the proliferation of smooth muscle cells by about 30 % with clinically relevant doses [130, 131]. Such a 30 % increase in prostate smooth muscle was considered likely to impede urination in males. The failure to consider the possibility of the hormetic response not only can lead to a lack of recognition of a desirable drug induced response but it can also result failure to prevent an adverse effect of drug treatment.

Of relevance to the present paper is that the hormetic dose response has been extensively reported across the spectrum of neuroscience research, including anxiolytic drugs [132], anti-seizure agents [133], pain [134] memory enhancing drugs [135], brain traumatic injury [136] several neurodegenerative diseases, including Alzheimer’s disease and Parkinson’s disease as well as for neurite outgrowth [137] and as astrocyte functioning [138]. Puzzo et al. [139] also reported that β-amyloid 25–35 enhanced LTP within a very detailed dose response study, clearly demonstrating an hormetic dose response. While numerous mechanisms have been shown to mediate hormetic processes [84], one common mechanism involved in affecting adaptive responses is the induction of broad spectrum of stress-related proteins, such as heat shock. The response features of these induced stress-related proteins typically follows an hormetic like biphasic dose response regardless of the inducing agent, affected cell type, and endpoint. For example, methyl mercury induced an hormetic biphasic dose response for GRP 78 protein expression (i.e., a marker of endoplastic reticulum stress) in the cerebral cortex of young adult male Sprague–Dawley rats [140]. The plant derived curcumin likewise induced a hormetic effect on proteasome activities in human keratinocytes [141]. The widely recognized cytoprotective heat shock protein inducing hydroxylamine derivative, bimoclomol, also induced adaptive hormetic responses in multiple systems involving a spectrum of neuropathologies [142]. Similar hormetic patterns are very general, extending from plants [143, 144] to invertebrates [145–147] and to mammals [148–151] affecting multiple indices of health and disease with a wide range of inducing agents.

Heat shock also induced protection in multiple organs including the brain with pre-conditioning protocols [152–155]. This is of particular relevance to the present paper because pre-conditioning represents a specific type of hormesis with respect to study design while still retaining its complete set of dose response features. These collective findings demonstrate that inducers of heat shock protein often follow an hormetic dose response pattern, and that observed optimized protection occurs in association with the maximal production of the heat shock protein response [145] and that this response can be abolished by specific pathway inhibitors, blocking the protection, thereby providing an explanatory mechanism for the hormetic protective effect.

## Theoretical bases and technical approaches

### Redox proteomic studies

Redox proteomics is the subset of proteomics in which oxidatively or nitrosatively modified proteins are identified, these are post-translational events that occur in the cell resulting in post-translational modification (PTM) of proteins.

The protein post translational modifications (PTM) play a crucial role in modifying the end product of expression and contribute towards biological processes and diseased conditions. Important posttranslational modifications include phosphorylation, acetylation, glycosylation, ubiquitination, and nitration [156]. The analysis of posttranslational modifications on a proteome scale is still considered an analytical challenge [157] because of the extremely low abundance of modified proteins among very complex proteome samples.

Among several types of post-translational protein modifications, phosphorylation and oxidative modifications play essential roles in the regulation of a variety of cell functions. Increasing evidence suggests that changes in protein modifications over time correlate with particular phenotypes and disease states. Reactions of free radicals and reactive oxygen and nitrogen species (ROS and RNS) with proteins lead to oxidative modifications such as formation of protein hydroperoxides, hydroxylation of aromatic groups and aliphatic amino acid side chains, nitration of aromatic amino acid residues, oxidation of sulfhydryl groups, oxidation of methionine residues, conversion of some amino acid residues into carbonyl groups, cleavage of the polypeptide chain and formation of cross-linking bonds. If oxidized proteins are not appropriately repaired or removed from cells, they are often toxic and can threaten cell viability [158]. Numerous studies demonstrated the harmful effects of irreversible oxidative PTM as a result of oxidative stress and increased levels of oxidatively-modified proteins have been shown to correlate with ageing [159]. Oxidative modifications of proteins lead to loss of their function, enzymatic activity, accumulation and inhibition of their degradation. All these metabolic dysfunctions have been observed in several human degenerative diseases such as cancer [160] and neurodegeneration [161]. Based on these findings, growing interest is currently given to better understand selective protein target of oxidative damage. Common markers of oxidative stress are: protein oxidation indexed by protein carbonyls, 3-nitrotyrosine (3-NT) and protein glutathionylation [162], lipid peroxidation indexed by thiobarbituric acid-reactive substances (TBARS), free fatty acid release, iso- and neuro-prostane formation, 2-propen-1-al (acrolein), and 4-hydroxy-2-transnonenal (HNE), DNA oxidation (8-hydroxy-2-deoxyguanosine) and advanced glycation end products (AGE) detection. In the case of increased oxidative stress, redox proteomics analyses are broadly used as a tool to identify proteins that are modified by ROS/RNS that contribute to the development of neurodegenerative [163–165] and other diseases. Recently, redox proteomics approach has been employed to identify post-translational modification of proteins caused by oxidative damage. Among general types of protein modifications, we focused our attention on protein carbonyls, lipid peroxidation adducts (HNE-adducts), glutathionylation and nitration of tyrosine residues (3-NT). An interesting bridge between HSP and PTM and redox proteomics has been found in the brain of subjects with AD. In the hippocampus of these individuals, oxidation and nitration of HO-1 have been linked to loss of function in terms of neuroprotection secondary to the significant reduction of BR production and BVR-ERK interaction [76]. The lines of evidence regarding the PTM on HO-1 are reinforced by the concomitant findings on the PTM on BVR’s structure [75, 166] thus corroborating the idea of an intriguing puzzle involving HSP and proteomics.

### Protein carbonylation

Carbonylation is an irreversible, non-enzymatic modification of proteins leading to a change in their activity or function. While protein carbonylation and the chemistry of the reactions that give rise to carbonyl groups are now well characterized [165], the overall biology of oxidative protein modifications is a complex picture and remains poorly defined. In more detail, protein carbonylation generated by reactive carbonyl species (RCS) arising by peroxidation of polyunsaturated fatty acids (PUFAs) has recently gained an even greater importance, in view of the emerging deleterious role of the RCS–protein adducts in the etiology and/or progression of several human diseases, such as cardiovascular (atherosclerosis, long-term complications of diabetes) and neurodegenerative diseases (AD, PD and cerebral ischemia). Most of the biological effects of intermediate RCS are due to their capacity to react with the nucleophilic sites of proteins, binding to the sulfhydryl group of cysteine (Cys), the ε-amino group of lysine (Lys) or the imidazole group of histidine (His) residues to form Michael or Schiff base protein adducts, known as advanced lipoxidation end-products (ALEs) [167]. As a consequence, determination of the biochemical factors that induce cellular responses resulting from protein carbonylation is now considered a key element to developing therapeutic approaches and ameliorating disease pathologies [168].

Indeed, proteins can be modified through the reaction between arginine and lysine amino groups with reducing sugars or reactive aldehydes, such as glyoxal and methylglyoxal, based on the Maillard reaction. This reaction is named glycation or non-enzymatic glycosylation. Glycation, which leads to the formation of early stage glycation adducts and then advanced glycation end products (AGE), is considered as one of the major cause of spontaneous damage to cellular and extracellular proteins [169]. Formation of AGEs on proteins is found in many tissues and is thought to contribute to a variety of age-associated diseases, such as AD and PD [170]. Although glycation is not an oxidative phenomenon, it can be amplified by oxidative stress and hence referred as to glycoxidation [171]. Thus, under conditions of oxidative stress, reducing sugars (glucose, fructose) and ascorbic acid can be self-oxidizing generating highly reactive dicarbonyl compounds, which can in turn react with proteins to form AGEs. Finally, among the most important glycating agents are the dicarbonyl compounds glyoxal and methylglyoxal that are derived from glucose auto-oxidation and glycolytic intermediates. The reactivity of these dicarbonyls is much higher than that of glucose, so they represent important precursors of AGEs in physiological systems. Interestingly, carbonylated adducts are being formed upon conjugation with these by dicarbonyl compounds. In addition, ROS can oxidize membrane lipids generating lipid hydroperoxides and many aldehydes such as acrolein, malonaldehyde, 4-hydroxy-2-nonenal (HNE) and other hydroxyalkenals.

### Lipid peroxidation adducts (HNE-adducts)

Lipid peroxidation is one of the major sources of free radical mediated injury that directly damages membranes and generates a number of secondary products. The overall process of lipid peroxidation consists of three stages: initiation, propagation and termination. Free-radical-mediated lipid peroxidation, occurs when a carbon-centred radical is produced on a PUFA (polyunsaturated fatty acid) by the abstraction of an allylic hydrogen by some form of radical present within the bilayer, for example the sulfuranyl free radical on Met^35^ ofA*β*-(1– 42). Oxygen, which lacks a dipole moment, diffuses into the lipid bilayer where it may react with the carbon-centred radical to form a lipid peroxyl-radical [172, 173]. The lipid peroxyl-radical may then abstract an allylic hydrogen from an adjacent polyunsaturated lipid which propagates the chain reaction and forms a lipid hydroperoxide that may then undergo cleavage forming an array of possible reactive aldehydes such as F2-isoprostane, HNE and 2-propenal (acrolein). HNE for example is primarily produced from arachidonic acid, an omega-6 PUFA with inflammatory and signalling properties of its own. In neuronal cells under A*β* toxicity an increase in HNE to concentrations of 5–10 *μ*M was demonstrated within the lipid bilayer [174]. HNE and malondialdehyde (MDA) are significantly elevated in several neurodegenerative diseases [175, 176]. HNE can accumulate in cells in relatively high concentrations and cause cell toxicity. Increased levels of HNE cause disruption of Ca2þ homeostasis, glutamate transport impairment, membrane damage, and cell death [177]. Once formed, HNE can covalently modify cysteine, lysine, or histidine residues by Michael addition altering protein structure and causing loss of function and activity [178]. Several methods have been developed for detection of free HNE, its metabolites, or its conjugation products with biomolecules. Other PUFAs that are important in the generation of lipid peroxidation products are LA (linoleic acid), DHA (docosohexanoic acid) and cholesterol, among others [173].

### Glutathionylation

One of the most common outcomes of a rise in cellular oxidant levels is the modification of redox-sensitive proteins via a process known as S-glutathionylation [179]. Glutathionylation occurs as a posttranslational modification of proteins at the cysteine residues by adding a glutathione (GSH, γ-glutamylcysteinylglycine) moiety [180]. Glutathione is a ubiquitous tripeptide that acts as an inherent antioxidant, and works in conjunction with oxidised glutathione (glutathione disulphide, GSSG) as an intracellular redox buffer. Glutathionylation can protect cysteine thiols against irreversible oxidation but can also alter, either positively or negatively, the activity of many proteins. Therefore, glutathionylation allows cells to sense and signal harmful stress conditions and trigger appropriate responses [181]. Protein disulfides are formed by the action of protein disulfide isomerase in the endoplasmic reticulum (ER). Inappropriate disulfide bridges (and cysteine sulfenic acids) arise in response to OS and are under the reversible control of the thioredoxin reductase system. Oxidatively damaged proteins have been reported as being cleared via ubiquitinylation followed by digestion in the 20S core of the 26S proteasome in the cytosol and nucleus [182]. Thus targeting ubiquitin bound to protein can be used as a parameter of protein oxidation. However, protein oxidation and ubiquitinylation might not be always directly correlated [183].

To date, a number of proteins have been identified that undergo S-glutathionylation, often in response to oxidative stress. S-Glutathionylation of proteins can induce a range of consequences [184], including a downregulation [185, 186] or upregulation [187] of enzymatic activity, altered DNA binding by transcription factors [188, 189], and increased [190] or decreased [191] protein stability.

### Nitration of tyrosine residues (3-NT)

3-NT (3-nitrotyrosine), a PTM of tyrosine, is a well-recognized marker of nitrosative stress. The formation of 3-NT is the product of the reaction between ONOO− and CO2 which form both NO2, a free radical, and the carbonate radical (CO3• −) through the intermediates nitrosoperoxycarbonate and nitrocarbonate [192]. However, there is evidence for alternative pathways, including one mediated by myeloperoxidase [193]. Nitration of tyrosine may also result in stearic hindrance that blocks a potential phosphorylation at the 4- paraposition, affecting the potential of tyrosine to be phosphorylated, resulting in a potential for decreased tyrosine signalling [152]. A decrease in tyrosine signalling may result in the progression of neurodegenerative diseases and increased 3-NT levels have been detected in AD, ALS, PD, atherosclerosis, and acute lung disease disorders [193, 194]. 3-NT formation has recently been linked to systemic autoimmune disorders such as lupus through the generation of endogenous antibodies against native proteins that may be nitrated [192].

### Redox proteomics methods

The complexity of tissue and body fluid proteomes calls for a separation step ahead of mass spectrometric (MS) analysis. Depending on the composition of the proteome, several well studied separation techniques are available including two-dimensional gel electrophoresis (2D-DE), liquid chromatography (LC) and capillary electrophoresis (CE) that are used in redox proteomics research into two major approaches; gel-based and non gel-based methods. With respect to the separation and mass spectrometric technique selected, proteins may have to be fractionated in a controlled manner into peptides through enzymatic digestion using e.g., trypsin up or downstream of the separation step.

### Gel–based methods

In gel-based methods, oxidized proteins extracted from biological tissues are separated using for example two-dimensional polyacrylamide gel electrophoresis (2-D PAGE) [195]. 2-D PAGE is currently one of the powerful protein separation method for the resolution of complex mixtures of proteins, permitting the simultaneous analysis of thousands of gene products. Proteins are separated according to their charge (pI) by isoelectric focusing (IEF) in the first dimension and according to their size (Mr) by SDS-PAGE in the second dimension. The introduction of immobilized pH gradients (IPG) for IEF has overcome the problems of pH gradient instability (caused by prolonged focusing time). IPGs allow the generation of pH gradients of any desired range (broad, narrow or ultra-narrow) between pH 3 and 12. In addition, the use of pH gradient allowed high riproducibility and large scale separations. However some limitations still remain to be solved including solubilization of membrane proteins, identification of low-abundance proteins and identification of highly idrofobic proteins. Coupled 2D-PAGE with immunochemical detection of protein carbonyl derivatized by 2,4-dinitrophenyhydrazine (DNPH), nitrated proteins indexed by 3-nitrotyrosine (3-NT), glyutathionylated proteins (GSH-bound proteins) and HNE-bound proteins followed by MS analysis is a workflow utilized in redox proteomics. Western blotting (WB) is usually incorporated in 2D-gel based redox proteomics because immunochemical detection of oxidatively modified proteins offers high sensitivity and specificity. A 2D western blot map is achieved by using specific antibodies, e.g., anti-DNP, anti-3-NT, anti-GSH or anti-HNE, that react with those proteins containing reactive carbonyl groups/3-NT/GSH/HNE. 2D gel images, used to obtain the protein expression profile, and the 2D western blots are analyzed by image software (PD Quest, BioRad). This sophisticated software offers powerful comparative analysis and is specifically designed to analyze many gels or blots at once that were performed under identical experimental conditions. Powerful automatching algorithms quickly and accurately match gels or blots and sophisticated statistical analysis tools identify experimentally significant spots. The principles of measuring intensity values by 2D analysis software are similar to those of densitometric measurements. After completion of spot matching, the normalized intensity of each protein spot from individual gels (or membranes) is compared between groups using statistical analysis [196].

Oxidized spots of interest are excised from the gel and identified using a peptide mass fingerprinting (PMF) MS approach [164, 197–199] The PMF approach can be carried out with matrix-assisted laser desorption ionization (MALDI)-MS or electrospray ionization (ESI)-MS. Gel-based methods are advantageous because they target a specific subset of the proteome and numerous gels can be ran and aligned with sophisticated software tools. Another used gel based redox proteomics method is two-dimensional difference gel electrophoresis (2D-DIGE). This method uses fluorescent dyes (e.g., Cy2, Cy3, and Cy5) to covalently label protein samples, allowing two to three samples to be analyzed simultaneously on the same gel in which signals are scanned at different wavelengths [200]. The common labeled residues are cysteine and lysine with functional NHS-ester and maleimides dyes, respectively. Incorporation of multiple dye molecules into protein sequence may change its migration significantly therefore minimal labeling has been developed to address this issue [201]. 2D-DIGE technique addresses the limitations of lack of reproducibility between gels and has femptomolar sensitivity and a 10^4^dynamic range [202].

### Non Gel-based methods

A non gel based proteomic method includes the digestion of proteins into peptides in solution, the nanoflow liquid chromatography (LC) separation or capillary electrophoresis (CE) of peptides and automated MS and MS/MS data acquisition. LC comprises high performance liquid chromatography (HPLC) or ultra high performance liquid chromatography (UHPLC) to achieve a high-resolution separation of various chemically different compounds depending on the LC column. Separation is achieved via differences in the affinities/distribution between the stationary and mobile phase. Modern nano LC systems achieve high resolution separation of peptides and are excellent tools for shot gun proteomics combined with data-dependent analysis [203]. Multi-dimensional protein identification technology (MudPIT) based on2-D LC allows the analysis of highly complex samples (tissues and body fluids) [204]. The high sensitivity associated with LC can also become a limitation towards interfering compounds. Sample carry-over is another limitation of LC characterised by the detection of residual analytes from previous measurements introducing biases into newer analyses [205]. Capillary electrophoresis perform separation of analytes from a complex protein mixture in a single step and with high resolution through buffer-filled capillaries flowing in a strong electrical field (300–500 V/cm). Additionally, CE–MS is fast, enabling separation of several thousand peptides in 60 min in a single run making it an ideal technology to be used in clinical proteomics [206]. A potential limitation is the fact that only small sample volumes can be applied to CE capillaries even if stacking approaches are used. In addition, the technique is not appropriate for the separation of proteins >20 kDa due to potential precipitation. With recent developments in mass spectrometry, such as isobaric tags for relative and absolute quantitation (iTRAQ) (Unwin et al.) selected reaction monitoring (SRM, which is also termed multiple reaction monitoring or MRM, [207], as well as stable isotope standards and capture by anti-peptide antibodies, the non-gel proteomic approach has become the method of choice because of its efficiency and convenience. Compared to the traditional gel-based method which is more complicated, the non-gel proteomic approach requiresonly 3 steps: digestionand labeling of resultant peptides, the separation of peptides and automated data acquisition.

Below, we report key examples of these recently reported non-gel proteomic approaches which hold the potential for identification of novel redox regulations associated with different disease. To quantify the level of the thiol modifications under oxidative stress conditions, thiol trapping techniques and isotope coded affinity tag (ICAT) can be combined to label unmodified and oxidized thiol groups with light and heavy tags, respectively [208]. This method named NOxICAT is specific for nitrosative and oxidative modifications of thiol groups. After protein denaturation, free thiol groups are labeled with light ICAT reagents. Oxidized thiols are labeled with heavy ICAT reagents and the extent of cysteine oxidation is measured by quantifying the relative ratios of light and heavy labeled peptides with MS. The Tandem Mass Tag(TMT) approach, which enables concurrent identification and multiplexed quantitation of proteins in different samples, is a well-established multiplex mass spectrometry analysis method. CysTMT is a version of the TMT approach that is thiol reactive [209]. The cysTMT reagents, which are several isobaric (mass and structure) isomers, can be used to label the sulfhydryl (−SH) groups irreversibly. CysTMT reagents react specifically with reduced cysteines in peptides and proteins. After labeling, peptides with various cysteine modifications, such as oxidation, disulfide bonds and S-nitrosylation can be identified and quantified by MS. Compared with the traditional biotin switch technique that has been widely used for identification of protein S-nitrosylation [210], this new reagent fulfills the requirements for a biotin switch label and offers some distinct advantages, including a permanent mass tag and the fragmentation of up to 6 isotopically balanced reporter ions between 126 and 131 Da permitting multiplex quantification [211]. Non-gel redox proteomics have been widely applied in the study of protein carbonylation. A key step in these approaches is the incorporation of enrichment procedures for protein carbonyl (PCO) which is necessary since the average abundance of carbonylated proteins has been reported as 0.2 % in human plasma [212]. Some reagents are able to directly increase the ionization efficiency of PCO prior to MS analysis. An example is dansylhydrazide which enhances efficiency of ionization due to its secondary nitrogens. Dansylhydrazide generates reproducible fragmentation patterns which allows MS3 scans to be employed for localization of PCO sites in proteins [213].

## Conclusions and perspectives

Although continually increasing resources are being expended to combat age-related diseases such as AD, PD, diabetes, osteoporosis, metabolic syndrome, and cancer, yet, the causal identification of these disease remain elusive. Thus, incidence and morbidity remain either constant or increase. Huge investments in biomedical research in the recent past have resulted in some striking accomplishments, including the sequencing of the human chromosomal single nucleotide polymorphisms (SNPs), and the identification of regional clusters of chromosomal SNPs (the HapMap). However, these accomplishments have failed to reveal the anticipated genetic causes for the common age-related diseases [214].

Modulation of endogenous cellular defense mechanisms via the stress response signaling represents an innovative approach to therapeutic intervention in diseases causing tissue damage, such as neurodegeneration, for example is reported how drugs that modulate proteostasis by inhibiting Hsp90 function or promoting Hsp70 function enhance the degradation of the critical aggregating proteins and ameliorate toxic symptoms in cell and animal disease models [215]. Efficient functioning of maintenance and repair processes seems to be crucial for both survival and physical quality of life. This is accomplished by a complex network of the so-called longevity assurance processes, which are composed of several genes termed vitagenes. Consistently, by maintaining or recovering the activity of vitagenes can be possible to delay the aging process and decrease the occurrence of age-related diseases with resulting prolongation of a healthy life span [2, 69, 216–218] As one of the most important neurodegenerative disorders, AD is a progressive disorder with cognitive and memory decline, speech loss, personality changes and synapse loss. With the increasingly aging population of the United States, the number of AD patients is predicted to reach 14 million in the mid-21st century in the absence of effective interventions. This will pose an immense economic and personal burden on the people of this country. There is now strong evidence to suggest that factors such as oxidative stress and disturbed protein metabolism and their interaction in a vicious cycle are central to AD pathogenesis. Brain-accessible antioxidants, potentially, may provide the means of implementing this therapeutic strategy of delaying the onset of AD, and more in general all degenerative diseases associated with oxidative stress. As one potentially successful approach, potentiation of endogenous secondary antioxidants systems can be achieved by interventions which target the HO-1/CO and/or Hsp70 systems.

The hormetic dose–response, challenges long-standing beliefs about the nature of the dose–response in a lowdose zone, having the potential to affect significantly the design of pre-clinical studies and clinical trials as well as strategies for optimal patient dosing in the treatment of numerous diseases [219, 220]. Reports exist of enhanced longevity via treatment with a large number of agents in a wide range of animal models displaying hormetic dose responses [25]. The generality of the hormetic dose response, being independent of biological model, endpoint, inducing agent and mechanism and with its quantitative features being a measure of plasticity constrained biological performance, strongly suggests that attempts to extend normal lifespan will be likewise limited to the 30–60 % as has been typically reported. Thus, hormesis has a fundamental role in aging research, affecting both the quality and the length of life as well as affecting the research methods (e.g., study design, statistical power, etc.) by which such biological concepts are studied.

Consisent to this notion, Protein redox regulation plays important roles in many biological processes. Protein cysteine thiols are sensitive to redox changes and may function as redox switches, which turn on or turn off signaling and metabolic pathways to ensure speedy responses to environmental stimuli or stresses. Novel integrative proteomics methods combining different isobaric tags in one experiment will permit simultaneous analysis of cysteine redox changes and total protein level changes, also allowing determination of redox modification in the cysteine pool in proteins. Thus, the capability to analyze protein posttranslational modification dynamics and protein level changes, particularly exploiting cellular stress proteome in one experiment will advance proteomic studies in many fields of biology and medicine.

## References

[1] Calabrese V, Cornelius C, Rizzarelli E, Owen JB, Dinkova-Kostova AT, Butterfield DA (2009). Nitric oxide in cell survival: a janus molecule. Antioxid Redox Signal.

[2] Calabrese V, Guagliano E, Sapienza M, Panebianco M, Calafato S, Puleo E (2007). Redox regulation of cellular stress response in aging and neurodegenerative disorders: role of vitagenes. Neurochem Res.

[3] Morimoto RI (2011). The heat shock response: systems biology of proteotoxic stress in aging and disease. Cold Spring Harb Symp Quant Biol.

[4] Calabrese V, Cornelius C, Mancuso C, Lentile R, Stella AM, Butterfield DA (2010). Redox homeostasis and cellular stress response in aging and neurodegeneration. Methods Mol Biol.

[5] Giffard RG, Macario AJ, de Macario EC (2013). The future of molecular chaperones and beyond. J. Clin. Invest.

[6] Morimoto RI, Cuervo AM (2014). Proteostasis and the aging proteome in health and disease. J Gerontol A Biol Sci Med Sci.

[7] Calabrese V, Cornelius C, Mancuso C, Barone E, Calafato S, Bates T (2009). Vitagenes, dietary antioxidants and neuroprotection in neurodegenerative diseases. Front Biosci.

[8] Calamini B, Silva MC, Madoux F, Hutt DM, Khanna S, Chalfant MA (2011). Small-molecule proteostasis regulators for protein conformational diseases. Nat Chem Bio.

[9] Kansanen E, Bonacci G, Schopfer FJ, Kuosmanen SM, Tong KI, Leinonen H (2011). Electrophilic nitro-fatty acids activate NRF2 by a KEAP1 cysteine 151-independent mechanism. J Biol Chem.

[10] Raynes R, Brunquell J, Westerheide SD (2013). Stress Inducibility of SIRT1 and Its Role in Cytoprotection and Cancer. Genes Cancer.

[11] Ryno LM, Genereux JC, Naito T, Morimoto RI, Powers ET, Shoulders MD (2014). Characterizing the altered cellular proteome induced by the stress-independent activation of heat shock factor 1. ACS Chem Biol.

[12] Kikis EA, Gidalevitz T, Morimoto RI (2010). Protein homeostasis in models of aging and age-related conformational disease. Adv Exp Med Biol.

[13] Leak RK (2014). Heat shock proteins in neurodegenerative disorders and aging. J Cell Commun Signal.

[14] Van Oosten-Hawle P, Morimoto RI (2014). Organismal proteostasis: role of cell-nonautonomous regulation and transcellular chaperone signaling. Genes Dev.

[15] Batulan Z, Taylor DM, Aarons RJ, Minotti S, Doroudchi MM, Nalbantoglu J (2006). Induction of multiple heat shock proteins and neuroprotection in a primary culture model of familial amyotrophic lateral sclerosis. Neurobiol Dis.

[16] Calderwood SK (2012). HSF1, a versatile factor in tumorogenesis. Curr Mol Med.

[17] Calderwood SK, Murshid A, Prince T (2009). The shock of aging: molecular chaperones and the heat shock response in longevity and aging--a mini-review. Gerontology.

[18] Gidalevitz T, Prahlad V, Morimoto RI. The stress of protein misfolding: from single cells to multicellular organisms. Cold Spring Harb Perspect Biol. 2011;3(6). doi:10.1101/cshperspect.a009704. 10.1101/cshperspect.a009704 PMC3098679 21536706

[19] Westerheide SD, Raynes R, Powell C, Xue B, Uversky VN (2012). HSF transcription factor family, heat shock response, and protein intrinsic disorder. Curr Protein Pept Sci.

[20] Broadley SA, Hartl FU (2009). The role of molecular chaperones in human misfolding diseases. FEBS Lett.

[21] Labbadia J, Morimoto RI (2014). Proteostasis and longevity: when does aging really begin?. F1000Prime Rep.

[22] Calabrese V, Cornelius C, Mancuso C, Pennisi G, Calafato S, Bellia F (2008). Cellular stress response: a novel target for chemoprevention and nutritional neuroprotection in aging, neurodegenerative disorders and longevity. Neurochem Res.

[23] Calabrese V, Calafato S, Cornelius C, Mancuso C, and Dinkova-Kostova. A Heme oxygenase: A master vitagene involved in cellular stress response. In: AM Eleuteri, editor. Enzymes and the Cellular Fight Against Oxidation. Research Signpost 2008, 37/661 (2), Fort P.O., Trivandrum-695 023, Kerala, India.

[24] Calabrese V, Cornelius C, Dinkova-Kostova AT, Calabrese EJ, Mattson MP (2010). Cellular stress responses, the hormesis paradigm, and vitagenes: novel targets for therapeutic intervention in neurodegenerative disorders. Antioxid Redox Signal.

[25] Calabrese V, Cornelius C, Dinkova-Kostova AT, Iavicoli I, Di Paola R, Koverech A (2012). Cellular stress responses, hormetic phytochemicals and vitagenes in aging and longevity. Biochim Biophys Acta.

[26] Calabrese V, Butterfield DA, Stella AM, Lajtha A, Perez-Polo JR, Rossner S (2008). Aging and oxidative stress response in the CNS. Development and Aging Changes in the Nervous System. Handbook of Neurochemistry and Molecular Neurobiology.

[27] Calabrese V, Cornelius C, Dinkova-Kostova AT, Calabrese EJ (2009). Vitagenes, cellular stress response and acetylcarnitine: relevance to hormesis. Biofactors.

[28] Mancuso C, Pani G, Calabrese V (2006). Bilirubin: An endogenous scavenger of nitric oxide and reactive nitrogen species. Redox Rep..

[29] Mancuso C, Barone E, Guido P, Miceli F, Di Domenico F, Perluigi M (2012). Inhibition of lipid peroxidation and protein oxidation by endogenous and exogenous antioxidants in rat brain microsomes in vitro. Neurosci Lett.

[30] Liu D, Gharavi R, Pitta M, Gleichmann M, Mattson MP (2009). Nicotinamide prevents NAD+ depletion and protects neurons against excitotoxicity and cerebral ischemia: NAD+ consumption by SIRT1 may endanger energetically compromised neurons. Neuromolecular Med.

[31] Liu DJ, Hammer D, Komlos D, Chen KY, Firestein BL, Liu AY (2014). SIRT1 knockdown promotes neural differentiation and attenuates the heat shock response. J Cell Physiol.

[32] Morimoto RI (2008). Proteotoxic stress and inducible chaperone networks in neurodegenerative disease and aging. Genes Dev.

[33] Morimoto RI, Santoro MG (1998). Stress-inducible responses and heat shock proteins: new pharmacologic targets for cytoprotection. Nat Biotechnol.

[34] Trovato Salinaro A, Cornelius C, Koverech G, Koverech A, Scuto M, Lodato F (2014). Cellular stress response, redox status, and vitagenes in glaucoma: a systemic oxidant disorder linked to Alzheimer’s disease. Front Pharmacol.

[35] Haslbeck M, Vierling E (2015). A first line of stress defense: small heat shock proteins and their function in protein homeostasis. J Mol Biol.

[36] Clerico EM, Tilitsky JM, Meng W, Gierasch LM (2015). How hsp70 molecular machines interact with their substrates to mediate diverse physiological functions. J Mol Biol.

[37] Macario AJ, Conway de Macario E (2007). Molecular chaperones: multiple functions, pathologies, and potential applications. Front Biosci.

[38] Gyurko DM, Soti C, Stetak A, Csermely P (2014). System level mechanisms of adaptation, learning, memory formation and evolvability: the role of chaperone and other networks. Curr Protein Pept Sci.

[39] Mattoo RU, Goloubinoff P (2014). Molecular chaperones are nanomachines that catalytically unfold misfolded and alternatively folded proteins. Cell Mol Life Sci.

[40] Clare DK, Saibil HR (2013). ATP-driven molecular chaperone machines. Biopolymers.

[41] Macario AJ, Conway de Macario E (2007). Chaperonopathies by Defect, Excess, or Mistake. Ann NY Acad Sci.

[42] Cortez L, Sim V. The therapeutic potential of chemical chaperones in protein folding diseases. Prion. 2014;8(2). Epub 2014 May 12. 10.4161/pri.28938 PMC4189890 24818993

[43] Hipkiss AR (2009). Error-protein metabolism and ageing. Biogerontology.

[44] Akude E, Zherebitskaya E, Chowdhury SK, Smith DR, Dobrowsky RT, Fernyhough P (2011). Diminished superoxide generation is associated with respiratory chain dysfunction and changes in the mitochondrial proteome of sensory neurons from diabetic rats. Diabetes.

[45] Muchowski PJ, Wacker JL (2005). Modulation of neurodegeneration by molecular chaperones. Nat Rev Neurosci.

[46] Baseler WA, Dabkowski ER, Williamson CL, Croston TL, Thapa D, Powell MJ (2011). Proteomic alterations of distinct mitochondrial subpopulations in the type 1 diabetic heart: contribution of protein import dysfunction. Am J Physiol Regul Integr Comp Physiol.

[47] Chowdhury SK, Dobrowsky RT, Fernyhough P (2011). Nutrient excess and altered mitochondrial proteome and function contribute to neurodegeneration in diabetes. Mitochondrion.

[48] Saibil HR (2013). Biochemistry. Machinery to reverse irreversible aggregates. Science.

[49] Priya S, Sharma SK, Goloubinoff P (2013). Molecular chaperones as enzymes that catalytically unfold misfolded polypeptides. FEBS Lett.

[50] Bersuker K, Hipp MS, Calamini B, Morimoto RI, Kopito RR (2013). Heat shock response activation exacerbates inclusion body formation in a cellular model of Huntington disease. J Biol Chem.

[51] Zhang K, Zhao T, Huang X, Liu ZH, Xiong L, Li MM (2009). Preinduction of HSP70 promotes hypoxic tolerance and facilitates acclimatization to acute hypobaric hypoxia in mouse brain. Cell Stress Chaperones.

[52] Delgado M, Varela N, Gonzalez-Rey E (2008). Vasoactive intestinal peptide protects against beta-amyloid-induced neurodegeneration by inhibiting microglia activation at multiple levels. Glia.

[53] Kakimura J, Kitamura Y, Takata K, Umeki M, Suzuki S, Shibagaki K (2002). Microglial activation and amyloid-beta clearance induced by exogenous heat-shock proteins. FASEB J.

[54] Siciliano R, Barone E, Calabrese V, Rispoli V, Butterfield DA, Mancuso C (2011). Experimental research on nitric oxide and the therapy of Alzheimer disease: a challenging bridge. CNS Neurol Disord Drug Targets.

[55] Bellia F, Vecchio G, Cuzzocrea S, Calabrese V, Rizzarelli E (2011). Neuroprotective features of carnosine in oxidative driven diseases. Mol Aspects Med.

[56] Brown IR (2007). Heat shock proteins and protection of the nervous system. Ann NY Acad Sci.

[57] Söti C, Csermely P (2007). Protein stress and stress proteins: implications in aging and disease. J Biosci.

[58] Kim HL, Cassone M, Otvos L, Vogiatzi P (2008). HIF-1alpha and STAT3 client proteins interacting with the cancer chaperone Hsp90: therapeutic considerations. Cancer Biol Ther.

[59] Muller P, Hrstka R, Coomber D, Lane DP, Vojtesek B (2008). Chaperone-dependent stabilization and degradation of p53 mutants. Oncogene.

[60] Okayama S, Kopelovich L, Balmus G, Weiss RS, Herbert BS, Dannenberg AJ (2014). p53 protein regulates Hsp90 ATPase activity and thereby Wnt signalling by modulating Aha1 expression. J Biol Chem.

[61] Mancuso C, Barone E (2009). The heme oxygenase/biliverdin reductase pathway in drug research and development. Curr Drug Metab.

[62] Maines MD (1997). The heme oxygenase system: a regulator of second messenger gases. Annu Rev Pharmacol Toxicol..

[63] Maines MD (1992). Heme Oxygenase in Clinical Applications and Functions.

[64] McCoubrey WK, Huang TJ, Maines MD (1997). Isolation and characterization of a cDNA from the rat brain that encodes hemoprotein heme oxygenase-3. Eur J Biochem.

[65] Maines MD, Panahian N (2001). The heme oxygenase system and cellular defense mechanisms. Do HO-1 and HO-2 have different functions?. Adv Exp Med Biol.

[66] Ryter SW, Choi AM (2013). Carbon monoxide: present and future indications for a medical gas. Korean J Intern Med.

[67] Mancuso C, Navarra P, Preziosi P (2010). Roles of nitric oxide, carbon monoxide, and hydrogen sulfide in the regulation of the hypothalamic-pituitary-adrenal axis. J Neurochem.

[68] Wu L, Wang R (2005). Carbon monoxide: endogenous production, physiological functions, and pharmacological applications. Pharmacol Rev.

[69] Mancuso C, Preziosi P, Grossman AB, Navarra P (1997). The role of carbon monoxide in the regulation of neuroendocrine function. Neuroimmunomodulation.

[70] Mancuso C, Scapagnini G, Currò D, Giuffrida Stella AM, De Marco C, Butterfield DA (2007). Mitochondrial dysfunction, free radical generation and cellular stress response in neurodegenerative disorders. Front Biosci..

[71] Mancuso C, Capone C, Ranieri SC, Fusco S, Calabrese V, Eboli ML (2008). Bilirubin as an endogenous modulator of neurotrophin redox signaling. J Neurosci Res.

[72] Barone E, Trombino S, Cassano R, Sgambato A, De Paola B, Di Stasio E (2009). Characterization of the S-denitrosylating activity of bilirubin. J Cell Mol Med.

[73] Stocker R (2004). Antioxidant activities of bile pigments. Antioxid Redox Signal.

[74] Minetti M, Mallozzi C, Di Stasi AM, Pietraforte D (1998). Bilirubin is an effective antioxidant of peroxynitrite-mediated protein oxidation in human blood plasma. Arch Biochem Biophys.

[75] Barone E, Di Domenico F, Cenini G, Sultana R, Coccia R, Preziosi P (2011). Oxidative and nitrosative modifications of biliverdin reductase-A in the brain of subjects with Alzheimer’s disease and amnestic mild cognitive impairment. J Alzheimers Dis.

[76] Barone E, Di Domenico F, Sultana R, Coccia R, Mancuso C, Perluigi M (2012). Heme oxygenase-1 posttranslational modifications in the brain of subjects with Alzheimer disease and mild cognitive impairment. Free Radic Biol Med.

[77] Barone E, Cenini G, Di Domenico F, Martin S, Sultana R, Mancuso C (2011). Long-term high-dose atorvastatin decreases brain oxidative and nitrosative stress in a preclinical model of Alzheimer disease: a novel mechanism of action. Pharmacol Res.

[78] Butterfield DA, Barone E, Di Domenico F, Cenini G, Sultana R, Murphy MP (2012). Atorvastatin treatment in a dog preclinical model of Alzheimer’s disease leads to up-regulation of heme oxygenase-1 and is associated with reduced oxidative stress in brain. Int J Neuropsychopharmacol.

[79] Barone E, Mancuso C, Di Domenico F, Sultana R, Murphy MP, Head E (2012). Biliverdin reductase-A: a novel drug target for atorvastatin in a dog pre-clinical model of Alzheimer disease. J Neurochem.

[80] Barone E, Di Domenico F, Mancuso C, Butterfield DA (2014). The Janus face of the heme oxygenase/biliverdin reductase system in Alzheimer disease: it’s time for reconciliation. Neurobiol Dis.

[81] Butterfield DA, Barone E, Mancuso C (2011). Cholesterol-independent neuroprotective and neurotoxic activities of statins: perspectives for statin use in Alzheimer disease and other age-related neurodegenerative disorders. Pharmacol Res.

[82] Abete P, Testa G, Cacciatore F, Della-Morte D, Galizia G, Langellotto A (2011). Ischemic preconditioning in the younger and aged heart. Aging Dis.

[83] Schulz H (1888). Uber Hefegifte. Pfluger’s Archiv Gesemmte Physiol.

[84] Calabrese EJ (2013). Hormetic mechanisms. Crit Rev Toxicol.

[85] Mitchel RE, Hasu M, Bugden M, Wyatt H, Hildebrandt G, Chen YX (2013). Low-dose radiation exposure and protection against atherosclerosis in ApoE(−/−) mice: the influence of P53 heterozygosity. Radiat Res.

[86] Blyth BJ, Azzam EI, Howell RW, Ormsby RJ, Staudacher AH, Sykes PJ (2010). An adoptive transfer method to detect low-dose radiation-induced bystander effects in vivo. Radiat Res.

[87] Phan N, Boreham DR (2011). Health effects from low dose occupational and medical radiation exposure and the role of adaptive response. Health Phys.

[88] Mothersill C, Seymour C (2014). Implications for human and environmental health of low doses of ionising radiation. J Environ Radioact.

[89] Nomura T, Sakai K, Ogata H, Magae J (2013). Prolongation of life span in the accelerated aging klotho mouse model, by low-dose-rate continuous γ irradiation. Radiat Res.

[90] Scott BR (2014). Radiation-hormesis phenotypes, the related mechanisms and implications for disease prevention and therapy. J Cell Commun Signal.

[91] Elmore E, Lao XY, Kapadia R, Swete M, Redpath JL (2011). Neoplastic transformation in vitro by mixed beams of high-energy iron ions and protons. Radiat Res.

[92] Calabrese EJ (2013). Origin of the linearity no threshold (LNT) dose–response concept. Arch Toxicol.

[93] Calabrese EJ (2013). Low doses of radiation can enhance insect lifespans. Biogerontology.

[94] Calabrese EJ (2010). Hormesis and homeopathy: introduction. Hum Exp Toxicol.

[95] Calabrese EJ, Calabrese V (2013). Low dose radiation therapy (LD-RT) is effective in the treatment of arthritis: animal model findings. Int J Radiat Biol.

[96] Calabrese EJ, Iavicoli I, Calabrese V (2013). Hormesis: its impact on medicine and health. Hum Exp Toxicol.

[97] Calabrese EJ (2013). Historical foundations of wound healing and its potential for acceleration: doseresponse considerations. Wound Repair Regen.

[98] Stebbing AR (2009). Interpreting ‘dose-response’ curves using homeodynamic data: with an improved explanation for hormesis. Dose Response.

[99] Sagan LA (1989). On radiation, paradigms, and hormesis. Science.

[100] Calabrese EJ (2015). Cancer risk assessment: Optimizing human health through linear dose–response models. Food Chem Toxicol.

[101] Luckey TD (2006). Radiation hormesis: the good, the bad, and the ugly. Dose Response.

[102] Thong H-Y, Maibach HI (2008). Hormesis [biological effects of low level exposure (BELLE)] and dermatology. Dose-Response.

[103] Calabrese V, Scapagnini G, Davinelli S, Koverech G, Koverech A, De Pasquale Salinaro AT (2014). Sex hormonal regulation and hormesis in aging and role of vitagenes. J Cell Commun Signal.

[104] Eaton DL and Klaassen CD. Principles of toxicology. In: Casarett & Doull’s Essentials of Toxicology, Chapter 2. The McGraw-Hill Companies, Inc. pp. 6–20.

[105] Calabrese V, Butterfield DA, Stella AM, Lajtha A, Perez-Polo JR, Rossner S (2008). Aging and oxidative stress response in the CNS. Development and Aging Changes in the Nervous System. Handbook ofNeurochemistry and Molecular Neurobiology.

[106] Calabrese EJ, Dhawan G, Kapoor R, Iavicoli I, Calabrese V. What is hormesis and its relevance to healthy aging and longevity? Biogerontology. 2015;16(6):693-707 10.1007/s10522-015-9601-0 26349923

[107] Calabrese EJ (1999). Evidence that hormesis represents an “overcompensation” response to a disruption in homeostasis. Ecotoxicol Environ Saf.

[108] Calabrese EJ, Baldwin LA (2000). Chemical hormesis: Its historical foundations as a biological hypothesis. Hum Exper Toxicol.

[109] Calabrese EJ (2013). Hormesis: Toxicological foundations and role in aging research. Exp Gerontol.

[110] Calabrese EJ, Blain RB (2009). Hormesis and plant biology. Environ. Poll..

[111] Calabrese EJ, Baldwin LA (2003). The hormetic dose response model is more common than the threshold model in toxicology. Tox Sci.

[112] Calabrese EJ, Baldwin LA (2003). Ethanol and hormesis. Crit Rev Toxicol.

[113] Calabrese EJ, Bachmann KA, Bailer AJ, Bolger PM, Borak J, Cai L (2007). Biological stress response terminology: Integrating the concepts of adaptive response and preconditioning stress within a hormetic dose–response framework. Toxicol Appl Pharmacol.

[114] Rattan SIS (2005). Hormetic modulation of aging and longevity by mild heat stress. Dose Response.

[115] Rattan SIS (2010). Targeting the age-related occurrence, removal, and accumulation of molecular damage by hormesis. Ann N Y Acad Sci.

[116] Rattan SIS, Ali RE (2007). Hormetic prevention of molecular damage during cellular aging of human skin fibroblasts and keratinocytes. Ann N Y Acad Sci.

[117] Rattan SIS, Gonzalez-Dosal R, Nielsen ER, Kraft DC, Weibel J, Kahns S (2004). Slowing down aging from within: Mechanistic aspect of anti-aging hormetic effects of mild heat stress on human cells. Acta Biochimica Polonica.

[118] Sarup P, Sorensen P, Loeschcke V (2014). The long-term effects of a life-prolonging heat treatment on the Drosophila melanogaster transcriptome suggest that heat shock proteins extend lifespan. Exp Gerontol.

[119] Arumugam TV, Gleichmann M, Tang SC (2006). Hormesis/preconditioning mechanisms, the nervous system and aging. Ageing Res Rev.

[120] Mattson MP (2008). Hormesis and disease resistance: activation of cellular stress response pathways. Hum Exp Toxicol.

[121] Mattson MP, Chan SL, Duan WZ (2002). Modification of brain aging and neurodegenerative disorders by genes, diet, and behavior. Physiol Rev.

[122] Okun E, Mattson MP, Veasey SC (2009). Neuronal vulnerability to oxidative damage in aging. Oxidative Neural Injury Book Series: Contemporary Clinical Neuroscience.

[123] Flood JF, Smith GE, Cherkin A (1983). Memory retention – Potentiation of cholinergic drugcombinations in mice. Neurobiol Aging.

[124] Flood JF, Smith GE, Cherkin A (1985). Memory enhancement – Supra-additive effect of subcutaneous chlolinergic drug-combinations in mice. Psychopharmacology.

[125] Calabrese EJ (2008). Neuroscience and Hormesis: Overview and general findings. Crit Rev Toxicol.

[126] Calabrese EJ, Baldwin LA (2002). Hormesis and high risk groups. Reg. Tox. Pharm..

[127] Calabrese EJ (2005). Cancer biology and hormesis: Human tumore cell lines commonly display hormetic (biphasic) dose responses. Crit Rev Toxicol.

[128] Randall WA, Price CW, Welch H (1947). Demonstration of hormesis (increase in fatality rate) by penicillin. Am J Pub Health.

[129] Welch H, Price CW, Randall WA (1946). Increase in fatality rate of E. Typhosa for white mice by streptomycin. J Am Pharm.

[130] Abramowitz J, Dai C, Hirschi KK, Dmitieva RI, Doris PA, Liu L (2003). Ouabain- and marinobufagenin-induced proliferation of human umbilical vein smooth muscle cells and a rat vascular smooth muscle cell lines, A7r5. Circulation.

[131] Chueh S-C, Guh J-H, Chen J, Lai M-K, Teng C-M (2001). Dual effects of ouabain on the regulation of proliferation and apoptosis in human prostatic smooth muscle cells. J Urol.

[132] Calabrese EJ (2008). An assessment of anxiolytic drug screening tests: hormetic dose responses predominate. Crit Rev Toxicol.

[133] Calabrese EJ (2008). Modulation of the epileptic seizure threshold: implications of biphasic dose responses. Crit Rev Toxicol.

[134] Calabrese EJ (2008). Pain and U-shaped dose responses: occurrence, mechanisms, and clinical implications. Crit Rev Toxicol.

[135] Calabrese EJ (2008). Alzheimer’s disease drugs: an application of the hormetic dose-response model. Crit Rev Toxicol.

[136] Calabrese EJ (2008). Drug therapies for stroke and traumatic brain injury often display U-shaped dose responses: occurrence, mechanisms, and clinical implications. Crit Rev Toxicol.

[137] Calabrese EJ (2008). Enhancing and regulating neurite outgrowth. Crit Rev Toxicol.

[138] Calabrese EJ (2008). Astrocytes: adaptive responses to low doses of neurotoxins. Crit Rev Toxicol.

[139] Puzzo D, Privitera L, Palmeri A (2012). Hormetic effect of amyloid-beta peptide in synaptic plasticity and memory. Neurobiol Aging.

[140] Zhang Y, Lu R, Liu W, Wu Y, Qian H, Zhao X (2013). Hormetic effects of acute methylmercury exposure on GRP78 expression in rat brain cortex. Dose–response.

[141] Ali RE, Rattan SIS (2006). Curcumin’s biphasic hormetic response on proteasome activity and heat-shock protein synthesis in human keratinocytes. Ann NY Acad Sci.

[142] Nánási PP, Sarkozi S, Szigeti G, Jona I, Szegedi C, Szabo A (2000). Biphasic effect of bimoclomol on calcium handling in mammalian ventricular myocardium. Brit J Pharmacol.

[143] Wang CR, Tian Y, Wang XR, Yu HX, Lu XW, Wang C (2010). Hormesis effects and implicative application in assessment of lead-contaminated soils in roots of *Vicia faba* seedlings. Chemosphere.

[144] Xu X, Huang Z, Wang C, Zhong L, Tian Y, Li D (2015). Toxicological effects, mechanisms, and implied toxicity threshold in the roots of *Vicia faba* L. seedlings grown in copper-contaminated soil. Environ Sci Pollut Res.

[145] Baruah K, Norouzitallab P, Linayati L, Sorgeloos P, Bossier P (2014). Reactive oxygen species generated by a heat shock protein (Hsp) inducing product contributes to Hsp70 production and Hsp70-mediated protective immunity in *Artemia franciscana* against pathogenic vibrios. Dev Comp Immunol.

[146] Lagisz M, Hector KL, Nakagawa S (2013). Life extension after heat shock exposure: assessing meta-analytic evidence for hormesis. Age Res Rev.

[147] Hranitz JM, Abramson CI, Carter RP (2010). Ethanol increases HSP70 concentrations in honeybee (*Apis mellifera* L.) brain tissue. Alcohol.

[148] Damelin LH, Vokes S, Whitcutt JM, Damelin SB, Alexander JJ (2000). Hormesis: a stress response in cells exposed to low levels of heavy metals. Hum Exper Toxicol.

[149] Sutton DJ, Tchounwou PB, Ninashvili N, Shen E (2002). Mercury induced cytotoxicity and transcriptionally activates stress genes in human liver carcinoma (HepG2) cells. Int J Mol Sci.

[150] Shutoh Y, Takeda M, Ohtsuka R, Haishima A, Yamaguchi S, Fujie H (2009). Low dose effects of dichlorodiphenyltrichloroethane (DDT) on gene transcription and DNA methylation in the hypothalamus of young male rats: implication of hormesis-like effects. J Toxicol Sci.

[151] Li SQ, Wang DM, Shu YJ, Wan XD, Xu ZS, Li EZ (2013). Proper heat shock pretreatment reduces acute liver injury induced by carbon tetrachloride and accelerates liver repair in mice. J Toxicol Pathol.

[152] Joyeux M, Godin-Ribuot D, Patel A, Demenge P, Yellon DM, Ribuot C (1998). Infarct size-reducing effect of heat stress and a1 adrenoceptors in rats. Brit J Pharmacol.

[153] Joyeux M, Lagneux C, Bricca G, Yellon DM, Demenge P, Ribuot C (1998). Heat stress-induced resistance to myocardial infarction in the isolated heart from transgenic [(mREN-2)27] hypertensive rats. Cardio Res.

[154] Joyeux M, Arnaud C, Godin-Ribuot D, Demenge P, Lamontagne D, Ribuot C (2002). Endocannabinoids are implicated in the infarct size-reducing effect conferred by heat stress preconditioning in isolated rat hearts. Cardio Res.

[155] Patel HH, Hsu A, Gross GJ (2002). Attenuation of heat shock-induced cardioprotection by treatment with the opiate receptor antagonist naloxone. Am J Physiol Heart Circ Physiol.

[156] Mann M, Jensen ON (2003). Proteomic analysis of post-translational modifications. Nat Biotechnol.

[157] Zhou H, Watts JD, Aebersold R (2001). A systematic approach to the analysis of protein phosphorylation. Nat Biotechnol.

[158] Shelton MD, Mieyal JJ (2008). Regulation by reversible S-glutathionylation: molecular targets implicated in inflammatory diseases. Mol Cells.

[159] Chakravarti B, Chakravarti DN (2007). Oxidative modification of proteins: age-related changes. Gerontology.

[160] Valko M, Rhodes CJ, Moncol J, Izakovic M, Mazur M (2006). Free radicals, metals and antioxidants in oxidative stress-induced cancer. Chem Biol Interact.

[161] Butterfield DA, Abdul HM, Newman S, Reed T (2006). Redox proteomics in some age-related neurodegenerative disorders or models thereof. NeuroRx.

[162] Berlett BS, Stadtman ER (1997). Protein oxidation in aging, disease, and oxidative stress. J Biol Chem.

[163] Butterfield DA, Sultana R (2008). Redox proteomics: Understanding oxidative stress in the progression of age-related neurodegenerative disorders. Expert Rev Proteomics.

[164] Butterfield DA, Perluigi M, Reed T, Muharib T, Hughes CP, Robinson RA (2012). Redox proteomics in selected neurodegenerative disorders: From its infancy to future applications. Antioxid Redox Signal.

[165] Stadtman ER, Levine RL, Dalle-Donne I, Scaloni A, Butterfield A (2006). Chemical modification of proteins by reactive oxygen species. Redox proteomics: from protein modifications to cellular dysfunction and diseases.

[166] Barone E, Di Domenico F, Cenini G, Sultana R, Cini C, Preziosi P (2011). Biliverdin reductase-A protein levels and activity in the brains of subjects with Alzheimer disease and mild cognitive impairment. Biochimica et Biophysica Acta.

[167] Colzani M, Aldini G, Carini M (2013). Mass spectrometric approaches for the identification and quantification of reactive carbonyl species protein adducts. J Proteomics..

[168] Colzani M, Criscuolo A, De Maddis D, Garzon D, Yeum KJ, Vistoli G (2014). A novel high resolution MS approach for the screening of 4-hydroxy-trans-2-nonenal sequestering agents. J Pharm Biomed Anal..

[169] Baraibar MA, Ladouce R, Friguet B (2013). Proteomic quantification and identification of carbonylated proteins upon oxidative stress and during cellular aging. J Proteomics..

[170] Li J, Liu D, Sun L, Lu Y, Zhang Z (2012). Advanced glycation end products and neurodegenerative diseases: mechanisms and perspective. J Neurol Sci.

[171] Baynes JW, Gillery P (2014). Frontiers in research on the Maillard reaction in aging and chronic disease. Clin Chem Lab Med.

[172] Butterfield DA, Castegna A, Lauderback CM, Drake J (2002). Evidence that amyloid β-peptide-induced lipid peroxidation and its sequelae in Alzheimer’s disease brain contribute to neuronal death. Neurobiol. Aging.

[173] Shichiri M (2014). The role of lipid peroxidation in neurological disorders. J. Clin. Biochem. Nutr.

[174] Mark RJ, Lovell MA, Markesbery WR, Uchida K, Mattson MP (1997). A role for 4-hydroxynonenal, an aldehydic product of lipid peroxidation, in disruption of ion homeostasis and neuronal death induced by amyloid β-peptide. J. Neurochem..

[175] Subramaniam R, Roediger F, Jordan B, Mattson MP, Keller JN, Waeg G (1997). The lipid peroxidation product, 4-hydroxy-2-trans-nonenal, alters the conformation of cortical synaptosomal membrane proteins. J Neurochem.

[176] Sultana R, Butterfield DA (2009). Proteomics identification of carbonylatedand HNE-bound brain proteins in Alzheimer’s disease. Methods Mol Biol.

[177] Reed TT (2011). Lipid peroxidation and neurodegenerative disease. Free Radic Biol Med.

[178] Markesbery WR, Lovell MA (1998). Four-hydroxynonenal, a product of lipid peroxidation, is increased in the brain in Alzheimer’s disease. Neurobiol Aging.

[179] Groitl B, Jakob U (1844). Thiol-based redox switches. Biochim. Biophys. Acta.

[180] Ghezzi P (2005). Oxidoreduction of protein thiols in redox regulation. Biochem. Soc. Trans..

[181] Giustarini D, Rossi R, Milzani A, Colombo R, Dalle-Donne I (2004). Sglutathionylation: From redox regulation of protein functions to human diseases. J Cell Mol Med.

[182] Grune T, Reinheckel T, Davies KJ (1997). Degradation of oxidized proteins in mammalian cells. FASEB J.

[183] Sheehan D (2006). Detection of redox-based modification in two-dimensional electrophoresis proteomic separations. Biochem Biophys Res Commun.

[184] Ghezzi P (2005). Regulation of protein function by glutathionylation. Free Radic. Res..

[185] Petrushanko IY, Yakushev S, Mitkevich VA, Kamanina YV, Ziganshin RH, Meng X (2012). S-Glutathionylation of the Na, k-atpase catalytic α subunit is a determinant of the enzyme redox sensitivity. J. Biol. Chem..

[186] Fratelli M, Demol H, Puype M, Casagrande S, Eberini I, Salmona M (2002). Identification by redox proteomics of glutathionylated proteins in oxidatively stressed human t lymphocytes. Proc. Natl. Acad. Sci. USA.

[187] Cabiscol E, Levine RL (1996). The phosphatase activity of carbonic anhydrase III is reversibly regulated by glutathiolation. Proc. Natl. Acad. Sci. USA.

[188] Klatt P, Lamas S (2000). Regulation of protein function by S-glutathiolation in response to oxidative and nitrosative stress. Eur J Biochem.

[189] Pineda-Molina E, Klatt P, Vazquez J, Marina A, Garcia de Lacoba M, Perez-Sala D (2001). Glutathionylation of the p50 subunit of NF-kappaB: a mechanism for redox-induced inhibition of DNA binding. Biochemistry.

[190] Davis DA, Newcomb FM, Starke DW, Ott DE, Mieyal JJ, Yarchoan R (1997). Thioltransferase (glutaredoxin) is detected within HIV-1 and can regulate the activity of glutathionylated HIV-1 protease in vitro. J. Biol. Chem..

[191] Liang JN, Pelletier MR (1988). Destabilization of lens protein conformation by glutathione mixed disulfide. Exp. Eye Res..

[192] Ahsan H (2013). 3-Nitrotyrosine: a biomarker of nitrogen free radical species modified proteins in systemic autoimmunogenic conditions. Hum. Immunol..

[193] Castegna A, Thongboonkerd V, Klein JB, Lynn B, Markesbery WR, Butterfield DA (2003). Proteomic identification of nitrated proteins in Alzheimer’s disease brain. J Neurochem.

[194] Sultana R, Perluigi M, Butterfield DA (2006). Protein oxidation and lipid peroxidation in brain of subjects with Alzheimer’s disease: insights into mechanism of neurodegeneration from redox proteomics. Antioxid Redox Signal.

[195] Chait BT (2006). Chemistry. Mass spectrometry: bottom-up or top-down?. Science.

[196] Butterfield DA, Perluigi M, Sultana R (2006). Oxidative stress in Alzheimer’s disease brain: new insights from redox proteomics. Eur J Pharmacol.

[197] Wittmann-Liebold B, Graack HR, Pohl T (2006). Two-dimensional gelelectrophoresis as tool for proteomics studies in combination with protein identification by mass spectrometry. Proteomics.

[198] Kim H, Eliuk S, Deshane J, Meleth S, Sanderson T, Pinner A (2007). 2D gel proteomics: An approach to study age-related differences in protein abundance or isoform complexity in biological samples. Methods Mol Biol.

[199] Sheehan D, McDonagh B, Barcena JA (2010). Redox proteomics. Expert Rev Proteomics.

[200] Unlu M, Morgan ME, Minden JS (1997). Difference gel electrophoresis: A single gel method for detecting changes in protein extracts. Electrophoresis.

[201] Gharbi S, Gaffney P, Yang A, Zvelebil MJ, Cramer R, Waterfield MD (2002). Evaluation of two-dimensional differential gel electrophoresis for proteomic expression analysis of a model breast cancer cell system. Mol Cell Proteomics.

[202] Timms JF, Cramer R (2008). Difference gel electrophoresis. Proteomics.

[203] Moruz L, Pichler P, Stranzl T, Mechtler K, Kall L (2013). Optimized nonlinear gradients for reversed-phase liquid chromatography in shotgun proteomics. Anal. Chem..

[204] Schirmer EC, Yates JR, Gerace L (2003). MudPIT: A powerful proteomics toolfor discovery. Discov. Med..

[205] Maes K, Smolders I, Michotte Y, Van EA (2014). Strategies to reduce aspecific adsorption of peptides and proteins in liquid chromatography-mass spectrometry based bioanalyses: an overview. J. Chromatogr. A.

[206] Stalmach A, Albalat A, Mullen W, Mischak H (2013). Recent advances in capillaryelectrophoresis coupled to mass spectrometry for clinical proteomic applications. Electrophoresis.

[207] Addona TA, Abbatiello SE, Schilling B, Skates SJ (2009). Multi-site assessment of the precision and reproducibility of multiple reaction monitoring- based measurements of proteins in plasma. Nat. Biotechnol..

[208] Lindemann C, Leichert LI (2012). Quantitative redox proteomics: the NOxICAT method. Methods Mol Biol..

[209] Thompson A, Schafer J, Kuhn K, Kienle S (2003). Tandem mass tags: a novel quantification strategy for comparative analysis of complex protein mixtures by MS/MS. Anal. Chem..

[210] Uehara T, Nakamura T, Yao D, Shi ZQ (2006). S-nitrosylated protein-disulphide isomerase links protein misfolding to neurodegeneration. Nature.

[211] Murray CI, Uhrigshardt H, O'Meally RN, Cole RN (2012). Identification and quantification of S-nitrosylation by cysteine reactive tandem mass tag switch assay. Mol. Cell. Proteomics.

[212] Madian AG, Regnier FE (2010). Proteomic identification of carbonylatedproteins and their oxidation sites. J Proteome Res.

[213] Palmese A, De Rosa C, Marino G, Amoresano A (2011). Dansyl labeling and bidimensional mass spectrometry to investigate protein carbonylation. Rapid Commun Mass Spectrom.

[214] Cornelius C, Perrotta R, Graziano A, Calabrese EJ, Calabrese V (2013). Stress responses, vitagenes and hormesis as critical determinants in aging and longevity: Mitochondria as a “chi”. Immun Ageing.

[215] Pratt WB, Gestwicki JE, Osawa Y, Lieberman AP (2015). Targeting Hsp90/Hsp70-Based Protein Quality Control for Treatment of Adult Onset Neurodegenerative Diseases. Annu Rev Pharmacol Toxicol.

[216] Calabrese V, Mancuso C, Ravagna A, Perluigi M, Cini C, De Marco C (2007). In vivo induction of heat shock proteins in the substantia nigra following L-DOPA administration is associated with increased activity of mitochondrial complex I and nitrosative stress in rats: regulation by glutathione redox state. J Neurochem.

[217] Calabrese V (2007). Highlight Commentary on “Redox proteomics analysis of oxidatively 3 modified proteins in G93A–SOD1 transgenic mice—A model of 4 familial amyotrophic lateral sclerosis”. Free Radical Biol Med.

[218] Calabrese V, Mancuso C, Sapienza M, Puleo E, Calafato S, Cornelius C (2007). Oxidative stress and cellular stress response in diabetic nephropathy. Cell Stress Chaperones.

[219] Mancuso C, Santangelo R, Calabrese V (2013). The heme oxygenase/biliverdin reductase system: a potential drug target in Alzheimer s disease. J Biol Regul Homeost Agent.

[220] Currò M, Trovato-Salinaro A, Gugliandolo A, Koverech G, Lodato F, Caccamo D (2015). Resveratrol protects against homocysteine-induced cell damage via cell stress response in neuroblastoma cells. J Neurosci Res.

